# SMURF1 and SMURF2 directly target GLI1 for ubiquitination and proteasome-dependent degradation

**DOI:** 10.1038/s41420-024-02260-4

**Published:** 2024-12-18

**Authors:** Fabio Bordin, Gloria Terriaca, Adriano Apostolico, Annamaria Di Fiore, Faranak Taj Mir, Sara Bellardinelli, Francesca Bufalieri, Rosa Bordone, Francesca Bellardinilli, Giuseppe Giannini, Gianluca Canettieri, Lucia Di Marcotullio, Elisabetta Ferretti, Marta Moretti, Enrico De Smaele

**Affiliations:** 1 Department of Experimental Medicine, Sapienza University of Rome, Rome, Italy; 2 Department of Molecular Medicine, Sapienza University of Rome, Rome, Italy

**Keywords:** CNS cancer, Tumour-suppressor proteins, Ubiquitylation

## Abstract

The transcription factor GLI1 is the main and final effector of the Hedgehog signaling pathway, which is involved in embryonic development, cell proliferation and stemness. Whether activated through canonical or non-canonical mechanisms, GLI1 aberrant activity is associated with Hedgehog-dependent cancers, including medulloblastoma, as well as other tumoral contexts. Notwithstanding a growing body of evidence, which have highlighted the potential role of post translational modifications of GLI1, the complex mechanisms modulating GLI1 stability and activity have not been fully elucidated. Here, we present a novel role played by SMURF1 and SMURF2 in the suppression of the Hedgehog/GLI signaling pathway through a direct targeting of GLI1. Indeed, the two SMURFs can interact with GLI1, exploiting the proline rich regions present on GLI1 protein, and trigger its polyubiquitination and proteasomal degradation, leading to a suppression of the Hedgehog pathway activity and a reduction of Hh-dependent tumor cell proliferation. Overall, this study adds new relevance to a tumor suppressive role of SMURFs on the Hedgehog pathway and confers upon them the status of potential therapeutic tools, either in canonical or non-canonical Hedgehog pathway aberrant activation.

## Introduction

The Hedgehog/GLI (Hh/GLI) signaling pathway is evolutionarily conserved in both invertebrates and vertebrates, and plays a crucial role in embryonic development, adult tissue homeostasis and regeneration [1].

Hh signaling dysregulation can lead to aberrant phenotypic consequences during development and pathological phenomena in adulthood [2]. Furthermore, the Hh signaling has been documented to play a crucial role in both the initiation and progression of tumors [2, 3].

The Hh pathway is canonically initiated by the interaction between a Hh ligand, and the transmembrane receptor Patched 1 (PTCH1) [1]. This binding relieves PTCH1-inhibition on the Smoothened (SMO) coreceptor, thereby triggering a cascade of events that leads to the activation of the transcription factors GLI1, GLI2, and GLI3. Subsequently, these factors translocate to the nucleus, orchestrating the expression of various genes associated with cell proliferation, survival, and angiogenesis [4].

The Hh pathway can be activated also through non-canonical mechanisms, via cross-talk with other signaling pathways such as TGFβ, KRAS, and WNT/β-catenin, which can upregulate GLI1 and/or GLI2 transcriptional activity [5]. These non-canonical pathways justify the potential relevance of the Hh/GLI pathway in tumoral contexts traditionally considered non Hh-dependent [6].

Among GLI proteins, GLI1 is considered the main effector of the Hh/GLI signaling, since it is also a transcriptional target of the pathway and provides an amplification loop which increases the signal [7]. Aberrant activation of GLI1 is indeed associated with numerous human cancers, including glioma, medulloblastoma (MB), osteosarcoma, rhabdomyosarcoma and colorectal cancer [6, 8].

Given the central role played by GLI1 in Hh-dependent tumorigenesis, it represents a promising therapeutic target. Indeed, targeting the final effectors of the signaling would allow therapy to be applied regardless of which pathway (canonical or non-canonical) has triggered their activation. This approach would circumvent the effects of mutations upstream of the pathway which in many cases provide resistance to therapies, such as the previously described targeting of the SMO receptor [9].

It is well known that GLI1 activity can be modulated by post-translational modifications (PTMs) of the protein [10]. Among PTMs, acetylation/deacetylation has a significant role, and the histone deacetylase HDAC1 has been demonstrated to deacetylate lysine 518 of GLI1, activating its translocation into the nucleus [11, 12]. On the other hand, it is known the involvement of ubiquitination in modulation of Hh/GLI, affecting the stability and homeostasis of GLI family proteins as well as other key players such as PTCH1, SMO, and HDAC1 [13]. An example of this regulation is the functional cooperation between NUMB and the HECT-E3 Ubiquitin Ligase ITCH, which leads to GLI1 ubiquitination and subsequent proteasome-dependent degradation [14, 15].

SMURF1 and SMURF2 proteins are two closely related HECT-E3 Ubiquitin Ligases belonging to the NEDD4 subfamily, the same as ITCH. SMURFs have been originally identified as negative regulators of the bone morphogenetic protein (BMP) and the transforming growth factor beta (TGF-β) pathways [16], but increasing evidence suggests the involvement of SMURF proteins in Hh pathway modulation. While in *Drosophila Melanogaster* the unique SMURF protein regulates the turnover of both PTCH1 and SMO [17], in mammals, SMURF1 and SMURF2’ role appears to be somewhat different.

In fact, SMURFs have been suggested to play a role in ubiquitination and lysosomal degradation of the PTCH1 receptor also in mammals [18] inducing an activation of the pathway, while SMURFs’ interaction on mammalian SMO has been ruled out [18]. More recently, it has been proposed that SMURF proteins induce ubiquitination and degradation of the RING Finger protein RNF220, which in turn targets the Embryonic Ectoderm Development (EED) protein, a component of the Polycomb Repressive Complex 2 (PRC2) [19]. Loss of SMURF would increase RNF220 protein and reduce PRC2 repression on the promoters of Hh targets (including GLI1) [20].

Structural and functional similarities between SMURFs and ITCH prompted us to investigate the hypothesis that SMURF1 and SMURF2 may also be able to act directly targeting GLI1.

Indeed, we report here that SMURF proteins can negatively regulate the Hh pathway through direct interaction with GLI1, inducing its ubiquitination and subsequent proteasomal degradation. Loss of GLI1, in turn, leads to a suppression of the Hh pathway and a significant reduction in Hh-dependent tumor cell proliferation.

The relevance of this newly described regulatory mechanism is also highlighted by the inverse correlation between *Smurfs* and *Gli1* expression in human MB expression profiling.

Our observations add new insights into the role of SMURFs on the Hh pathway, identifying them as direct negative regulators of GLI1, the main effector of the signaling, and confers upon them the status of promising therapeutic target, both in canonical and non-canonical Hh pathway activation.

## Results

### SMURF proteins overexpression reduces GLI1 protein levels

**Fig. 1:**
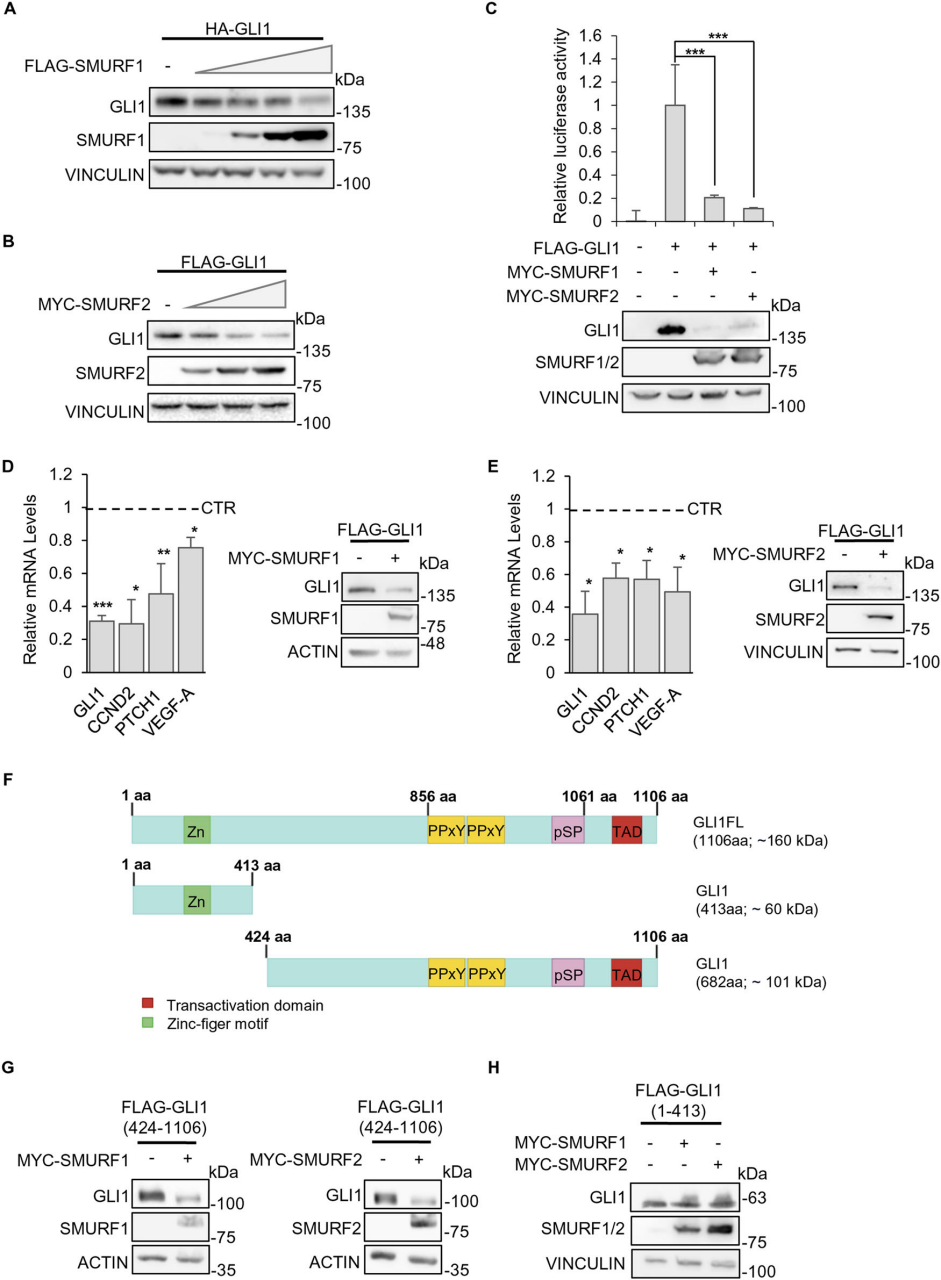
SMURFs overexpression reduces GLI1 protein levels. **A**, **B** Effect of SMURFs overexpression on GLI1 protein levels. HEK-293T cells co-expressing HA-GLI1 or FLAG-GLI1 plasmids and increasing amounts of either FLAG-SMURF1, MYC-SMURF2 or control vector. 24 h after transfection the cells were lysed, and proteins analysed using SDS-PAGE. Proteins were detected using antibodies anti-HA, anti-FLAG, anti-MYC and anti-VINCULIN (used as a normalizer). **C** Luciferase assay using a GLI1-RE activity with or without SMURF1/SMURF2 expression. HEK-293T cells were co-transfected with the vectors Gli1RE-12xLuc and pRL-TK-Renilla (used as a normalizer), along with various combinations of expression vectors FLAG-GLI1, MYC-SMURF1 or MYC-SMURF2 as indicated. 24 h after transfection the cells were lysed for protein expression and luciferase assay. The luciferase results are expressed as a Luciferase/Renilla ratio and were normalized to the control. Protein lysates were analysed using SDS-PAGE, and proteins were detected with antibodies anti-GLI1, anti-MYC and anti-VINCULIN (used as a normalizer). (*p < 0.05, **p < 0.01, ***p < 0.001; results are expressed as the mean ± SD of three independent experiments, Student’s *t*-test). **D**, **E** Analysis on Hh/GLI1 target genes expression, following SMURF1 or SMURF2 expression in HEK-293T cells. 24 h after transfection the cells were lysed for protein and mRNA extraction. mRNA levels of *Gli1*, *Cyclin D2, Ptch1* and *VEGF-A* were analysed by RT-qPCR and normalized to the average of three housekeeping genes. Protein lysates were analysed by SDS-PAGE, and proteins were detected with antibodies anti-FLAG, anti-MYC, anti-ACTIN and anti-VINCULIN (used as normalizers). (*p < 0.05, **p < 0.01; results are expressed as the mean ± SD of three independent experiments, Student’s *t*-test). **F** Schematic representation of structure and domains of full-length(FL) and truncated GLI1 protein. **G**, **H** Effect of SMURFs overexpression on protein levels of GLI1 truncated forms. HEK-293T cells co-expressing truncated FLAG-GLI1 (424-1106) or FLAG-GLI1 (1-413), MYC-SMURF1, MYC-SMURF2 or control vector were lysed, and the lysate was analysed using SDS-PAGE. Proteins were detected using antibodies anti-FLAG, anti-MYC, and anti-VINCULIN or anti-ACTIN (used as normalizers).

In order to verify the hypothesis of a direct effect of SMURFs Ubiquitin Ligases on GLI1, we transfected HEK-293T cells with plasmids encoding for a HA- or FLAG-tagged GLI1 and either SMURF1 or SMURF2. We observed that SMURFs overexpression leads to a dose-dependent decrease in GLI1 protein levels (Fig. 1A, B).

Similarly, using a GLI1-responsive luciferase reporter we demonstrated that reduction of exogenous GLI1 protein levels due to the expression of either SMURF1 or SMURF2 was followed by decreased reporter activity (Fig. 1C).

Consistent with these results, overexpression of SMURF1 or SMURF2 induced a reduction in endogenous mRNA levels of key transcriptional targets of the Hh/GLI pathway, including *Gli1* itself, the Hh-transmembrane receptor *Ptch1*, the cell cycle regulator *Cyclin D2* and the angiogenesis regulator *Vegf-a* (Fig. 1D, E).

It has been previously shown that GLI1 protein presents, between amino acids 856 and 1061, proline-containing PPxY and pSP motifs that can be recognized and bound by the WW domains of the ITCH Ubiquitin Ligase [15]. We therefore expressed truncated forms of GLI1, containing or not the PPxY/pSP motifs (Fig. 1F), and demonstrated that SMURF proteins act only on the PPxY/pSP containing fragment. In particular, we observed that overexpression of either SMURF1 or SMURF2 protein led to a decreased expression of the 424-1106 truncated form but not of the 1-413 fragment (Fig. 1G, H).

### SMURFs interact directly with GLI1 protein and induce its ubiquitination

Given that the action of SMURFs on GLI1 protein seems to depend on the presence of the proline rich motifs, which could be recognized and directly bound by SMURFs, we hypothesized a direct interaction between SMURFs and GLI1.

**Fig. 2:**
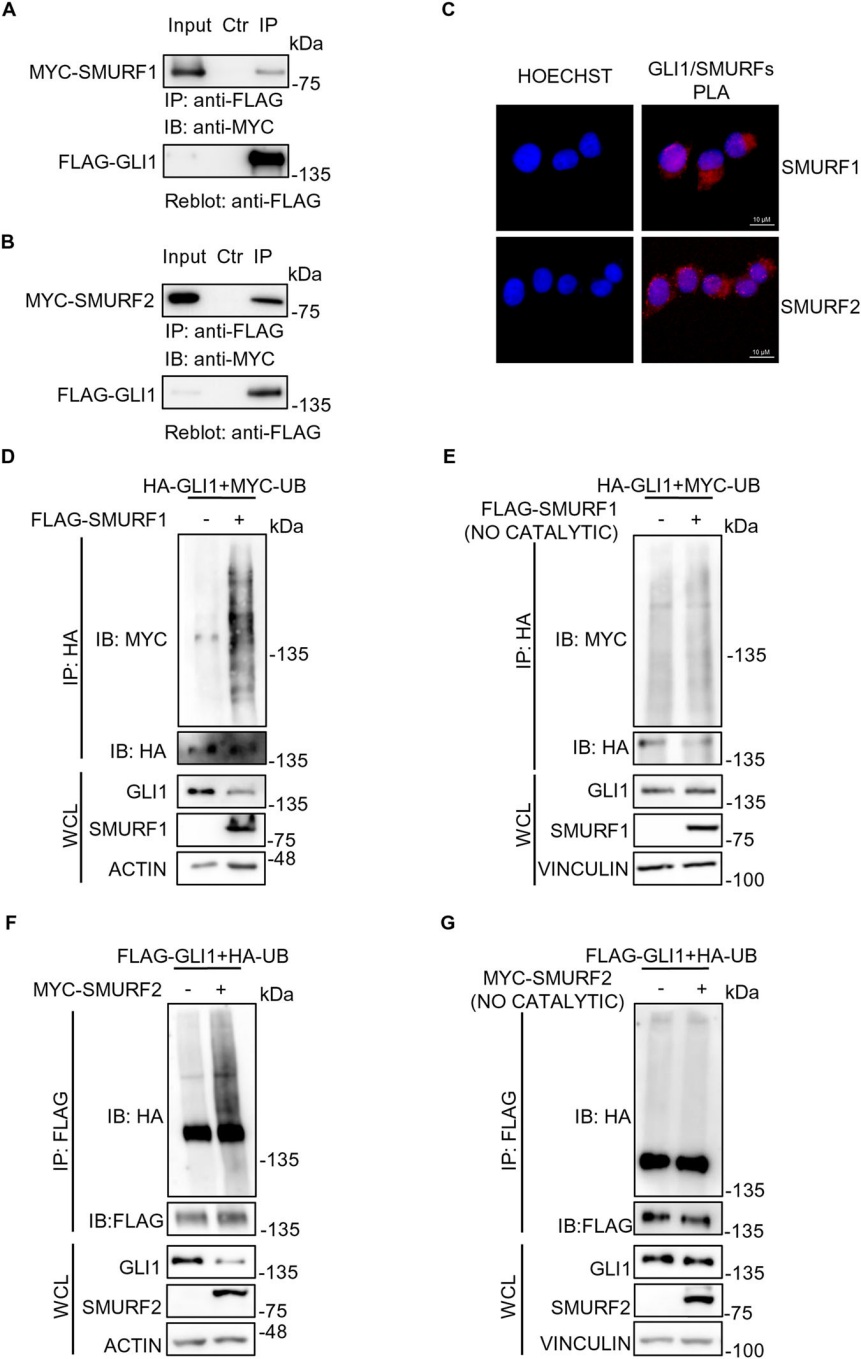
SMURF proteins interact with and increase the ubiquitination levels of full length GLI1 protein. **A**, **B** Co-IP assays on HEK-293T cells expressing FLAG-GLI1 and either MYC-SMURF1 or MYC-SMURF2. 24 h after transfection the cells were lysed, and Co-IP assay was performed using anti-FLAG conjugated agarose beads. As a control, the agarose beads were saturated with the FLAG peptide. Antibodies anti-FLAG and anti-MYC were used to detect the immunocomplexes. **C** Proximity ligation assay (PLA) for SMURFs and GLI1 interaction. HEK-293T cells were transfected with a construct codifying for FLAG-GLI1. 24 h after transfection, cells were fixed in paraformaldehyde (PFA) and permeabilized. Subsequently, cultures were processed with primary antibody anti-FLAG, anti-GLI1, anti-SMURF1 or anti-SMURF2, and with specific secondary antibodies for PLA assay (red signal), as described in Methods. Nuclei were stained blue (Hoechst). **D**, **G** Ubiquitination assays on HEK-293T cells co-expressing HA-GLI1 or FLAG-GLI1, in combination with exogenous Ubiquitin (MYC-UB or HA-UB), and either control vectors, FLAG-SMURF1 (catalytic or not catalytic) or MYC-SMURF2 (catalytic or not catalytic). 24 h after transfection, cells were lysed, and the ubiquitination status was evaluated by immunoprecipitation with anti-HA or anti-FLAG conjugated agarose beads. Proteins were detected using antibodies anti-FLAG, anti-HA, anti-MYC, and anti-ACTIN or anti-VINCULIN (used as a normalizer).

To verify this hypothesis, we performed Co-Immunoprecipitation (Co-IP) assays, observing that both SMURF1 and SMURF2 can form complexes with GLI1 (Fig. 2A, B).

The Co-IP data was further confirmed through Proximity ligation assays (PLA), which offers the opportunity to visualize protein-protein interaction, provided that the probes which recognize the target proteins are within 40 nm [21]. The assays, performed in HEK-293T cells, demonstrated that both endogenous SMURF1 and SMURF2 can interact with exogenous GLI1 (Fig. 2C). The same experiment was conducted also on Hh-responsive NIH-3T3 cells, which allowed us to confirm the interaction between endogenous proteins (Supplementary Fig. S1).

Given the well-known ubiquitination capability of SMURFs Ligases, and their interaction with GLI1, we performed ubiquitination assays following overexpression of SMURF1 and SMURF2 in HEK-293T cells. As expected, we observed an increase in the ubiquitination levels of GLI1 (Fig. 2D, F), which was lost when we expressed catalytically inactive mutants of SMURFs (Fig. 2E, G).

### SMURF proteins trigger proteasome-dependent degradation of GLI1 by promoting its K48-linked poly-ubiquitination

**Fig. 3:**
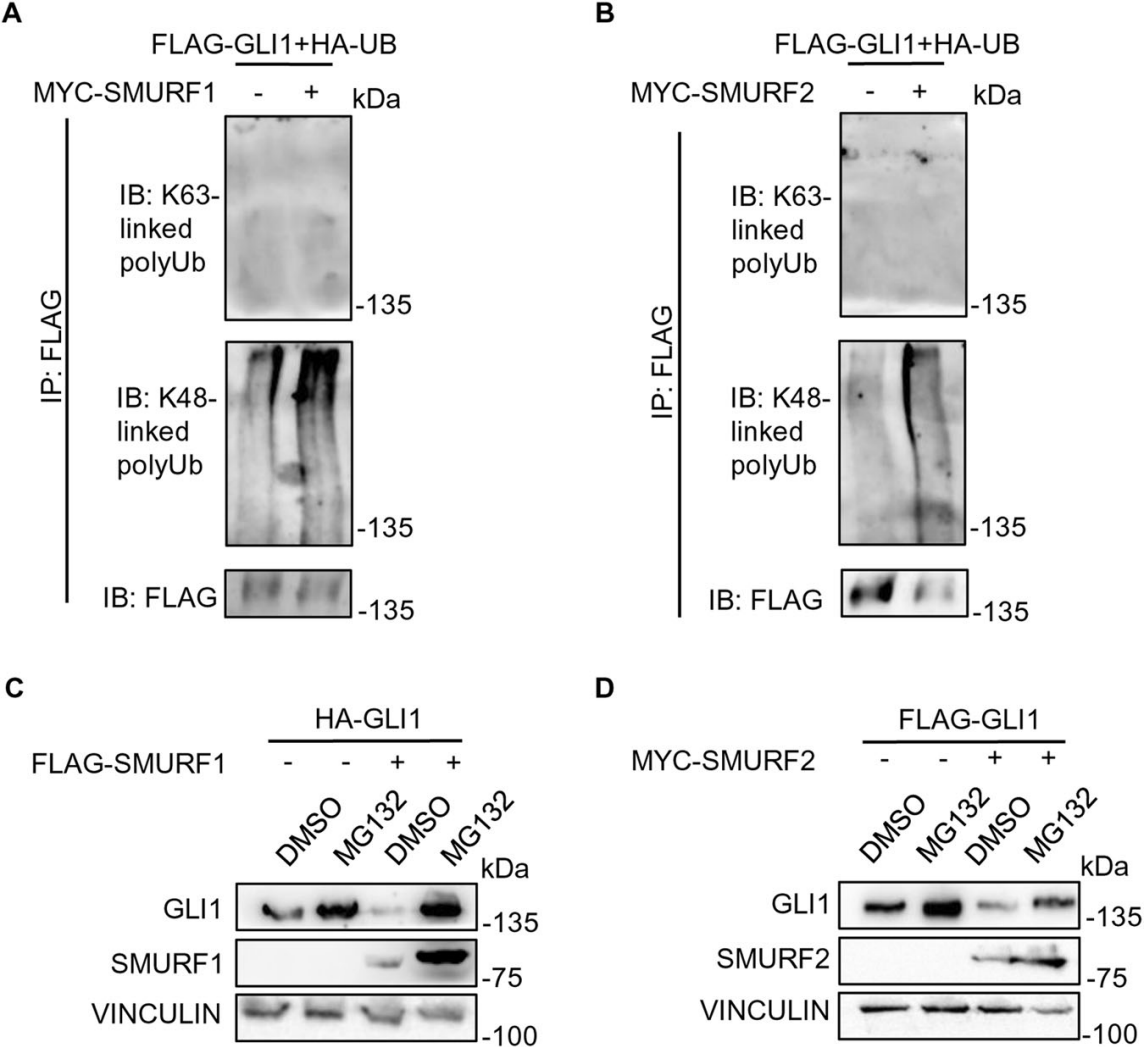
SMURF proteins mediate GLI1 K48-poly-ubiquitination, leading to GLI1 proteasomal degradation. **A**, **B** Ubiquitination assays of HEK-293T cells following expression of FLAG-GLI1, HA-UB and either control vectors, MYC-SMURF1 or MYC-SMURF2. 24 h after transfection, cells lysates were immunoprecipitated with FLAG agarose beads and analysed by SDS-PAGE. Proteins were detected using antibodies anti-FLAG, anti-MYC and specific antibodies against K48-linked/K63-linked poly-ubiquitination to detect the ubiquitination status of GLI1. **C**, **D** Analysis of GLI1 protein levels following SMURF1/SMURF2 expression in presence of proteasome inhibitor MG132. HEK-293T were transfected with HA-GLI1 or FLAG-GLI1, in combination with control vector, FLAG-SMURF1 or MYC-SMURF2. 24 h post transfection, cells were treated with DMSO or MG132 at 1 µM for 16 h. Subsequently, cells were lysed and analysed by SDS-PAGE. Proteins were detected using antibodies anti-FLAG, anti-HA, anti-MYC, and anti-VINCULIN (used as a normalizer).

Since HECT ligases catalyse both K48- or K63-linkage polyubiquitination, driving the target proteins to different fates [22] we analysed the type of ubiquitination triggered by SMURFs, using specific antibodies. Indeed, we observed a significant K48-linked polyubiquitination of GLI1, which targets the proteins to proteasomal degradation, while we did not detect K63-linked polyubiquitination, which mainly target proteins to lysosomal degradation [22] (Fig. 3A, B). Coherently, the proteasome inhibitor MG132 abolished the degradative effect induced by SMURF proteins on GLI1 (Fig. 3C, D).

### SMURFs direct interaction with the proline-rich regions of GLI1 is sufficient to induce its ubiquitination

**Fig. 4:**
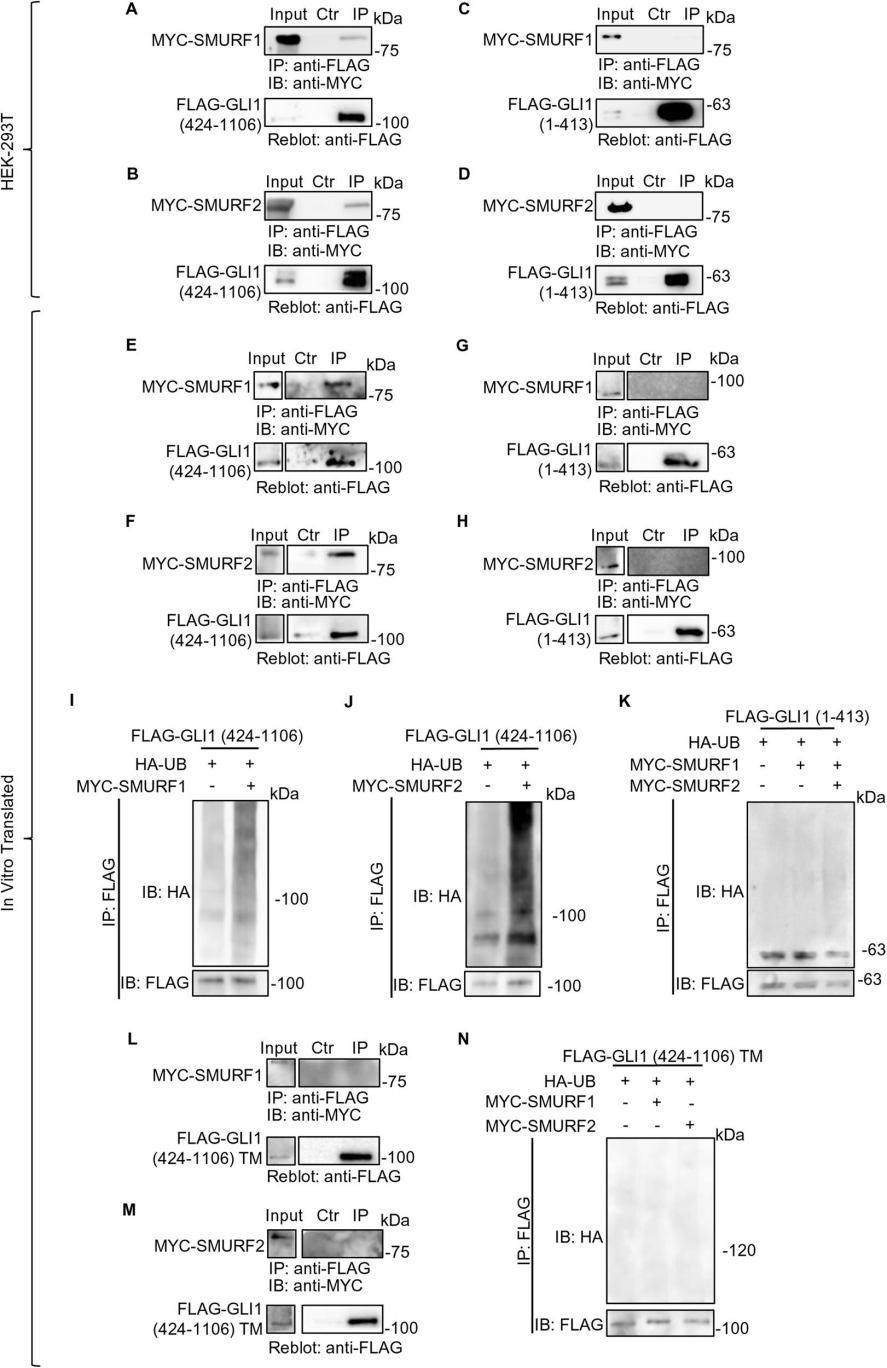
SMURF proteins interact with the 424-1106 region of GLI1, recognizing its proline rich motifs. Co-IP assays on HEK-293T cells expressing either MYC-SMURF1 or MYC-SMURF2, together with FLAG-GLI1 (424-1106; **A**, **B**) or FLAG-GLI1 (1-413; **C**, **D**). 24 h after transfection, cells lysates were used to perform Co-IP assay following immunoprecipitation with anti-FLAG conjugated agarose beads. In the control the agarose beads were saturated with the FLAG peptide. Anti-FLAG and anti-MYC antibodies were used to detect the immunocomplexes. Co-IP assays between in vitro translated (IVT) proteins. MYC-SMURF1 and MYC-SMURF2 together with FLAG-GLI1 (424-1106; **E**, **F**) or FLAG-GLI1 (1-413; **G**, **H**), were translated as described in Methods and immunoprecipitated using anti-FLAG conjugated agarose beads. In the control, the agarose beads were saturated with the FLAG peptide. Anti-FLAG and anti-MYC antibodies were used to detect the immunocomplexes. In vitro ubiquitination assays using IVT protein. IVT FLAG-GLI1 (424-1106; **I**, **J**) or FLAG-GLI1 (1-413; **K**) were immunoprecipitated by using anti-FLAG conjugated agarose beads and incubated with in vitro ubiquitination components (as indicated in Methods) in the presence of either MYC-SMURF1 or MYC-SMURF2 IVT proteins. Proteins were analysed by SDS-PAGE and detected using antibodies anti-FLAG, anti-MYC, and anti-HA. **L**, **M** Co-IP assays between IVT proteins. FLAG-GLI1 (424-1106) triple mutant (TM), MYC-SMURF1 and MYC-SMURF2 IVT proteins were used to perform Co-IP assay following immunoprecipitation with anti-FLAG conjugated agarose beads. In the control, the agarose beads were saturated with the FLAG peptide. Antibodies anti-FLAG and anti-MYC were used to detect the immunocomplexes. **N** In vitro ubiquitination assays using IVT proteins. FLAG-GLI1 (424-1106) TM IVT was immunoprecipitated by using anti-FLAG conjugated agarose beads and incubated with in vitro ubiquitination components, in the presence or absence of either MYC-SMURF1 or MYC-SMURF2 IVT proteins. Proteins were analysed by SDS-PAGE and detected using antibodies anti-FLAG, anti-MYC, and anti-HA.

To identify the region of GLI1 which is crucial for SMURFs interaction, we performed Co-IP assays with plasmids encoding GLI1 truncated forms (see Fig. 1F). Indeed, we observed that both SMURF1 and SMURF2 are capable to co-immunoprecipitate with the GLI1 truncated form 424-1106 (containing the proline-rich regions; Fig. 4A, B), but not with the 1-413 fragment (Fig. 4C, D).

To confirm the direct interaction between SMURFs and GLI1, we translated in vitro utilizing the cell-free rabbit reticulocyte lysate system SMURF1 and SMURF2, along with the two truncated variants of GLI1, and conducted Co-IP assays.

Even within the cell-free context, we observed that SMURF proteins were able to co-immunoprecipitate with the 424-1106 truncated form (Fig. 4E, F). As expected, SMURF proteins were unable to bind the GLI1 1-413 fragment (Fig. 4G, H), confirming the specificity of the observed interactions.

These observations allowed us to exclude the need for further mediators/adaptors in the protein complex formation, which have been suggested for other HECT Ubiquitin Ligases and their protein substrates [23].

Furthermore, we conducted cell-free ubiquitination assays using the in vitro translated proteins together with purified ubiquitination reaction components and confirmed the specific and direct activity of SMURF proteins on the GLI1 form 424-1106, while no significant ubiquitination was observed on the GLI1 1-413 fragment (Fig. 4I–K).

It has been previously demonstrated that mutation of the proline-rich domains present in the 424-1106 region of GLI1 abolished its interaction with the Ubiquitin Ligase ITCH [15]. Given the structural similarities between SMURF proteins and ITCH, we used a GLI1 mutant characterized by three-point mutations within the two PPXY and the pSP domains. Specifically, the GLI1 triple mutant (GLI1 TM) is characterized by two tyrosine replacing two phenylalanine residues at positions 859 and 872, and an alanine residue replacing a serine at position 1060 [15].

We performed Co-IP assays between GLI1 TM and SMURF proteins in vitro translated, observing SMURF1 and SMURF2 inability to interact with GLI1 TM (Fig. 4L, M). Consistently, in vitro ubiquitination shows unchanged ubiquitination levels of GLI1 TM in presence of either SMURF1 or SMURF2 (Fig. 4N).

### SMURFs modulation affects GLI1 ubiquitination and protein levels in MB tumour cells

Once clarified the regulatory mechanism of SMURF proteins on GLI1, we investigated their effect in MB cell lines, specifically using DAOY, ONS-76, UW-228, and D283. The first three have been characterized as Hh-dependent MB cell lines [24, 25], while D283 cells have been classified variably to groups 3 or 4 but still exhibit a certain level of GLI1 expression and activity [26, 27].

**Fig. 5:**
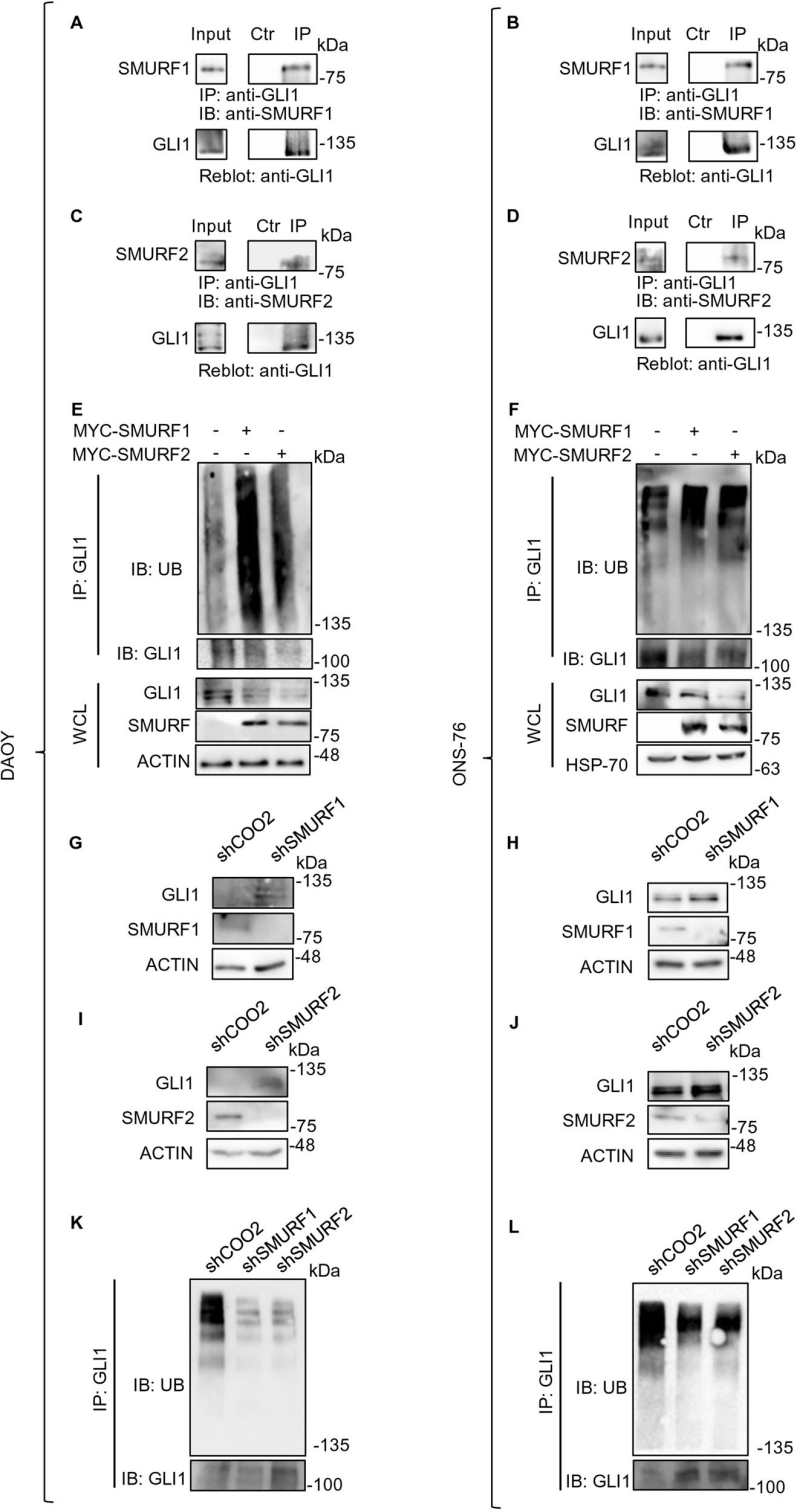
SMURFs modulation affects GLI1 ubiquitination and protein levels in MB cells. **A**, **D** Endogenous Co-IP assays on DAOY and ONS-76 cells. Cells were lysed and the lysate was immunoprecipitated with either a GLI1 antibody or a control IgG antibody. Antibodies anti-GLI1, anti-SMURF1 and anti-SMURF2 were used to detect the immunocomplexes. **E**, **F** Ubiquitination assays on DAOY and ONS-76 cells. Cells were transfected with either control vector, MYC-SMURF1 or MYC-SMURF2. 24 h after transfection, cells were lysed, and the ubiquitination status was evaluated following GLI1 immunoprecipitation. Proteins were detected using antibodies anti-GLI1, anti-SMURF1, anti-SMURF2, anti-Ubiquitin and anti-ACTIN (used as a normalizer) **G**–**J** Analysis of GLI1 protein levels following SMURFs silencing. DAOY and ONS-76 cells were stably transduced with the indicated shRNAs against SMURF1 or SMURF2. Cell lysates were analysed using SDS-PAGE. Proteins were detected using antibodies anti-GLI1, anti-SMURF1, anti-SMURF2 and anti-ACTIN (used as a normalizer). **K**, **L** Ubiquitination assays on DAOY and ONS-76 cells stably transduced with the indicated shRNAs against SMURF1 or SMURF2. The ubiquitination status was evaluated following GLI1 immunoprecipitation. Proteins were detected using antibodies anti-GLI1 and anti-Ubiquitin.

First, we verified the ability of endogenous SMURFs and GLI1 proteins to co-immunoprecipitate in a tumoral context. To this end, we performed Co-IP experiments and confirmed protein interaction in DAOY and ONS-76 (Fig. 5A–D).

Subsequently, we demonstrated that overexpression of either SMURF1 or SMURF2 leads to a reduction in endogenous GLI1 protein levels that is associated with an increase in GLI1 endogenous ubiquitination levels (Fig. 5E, F). The type of ubiquitination was the K48-linked polyubiquitination of GLI1, which is known to be associated with protein degradation, (Supplementary Fig. S2).

Conversely, silencing the expression of SMURF1 and SMURF2 using shRNA induced an increase in GLI1 protein levels in both MB cell lines (Fig. 5G–J) due to a reduction in endogenous GLI1 ubiquitination levels (Fig. 5K and L).The effects of SMURFs modulation on GLI1 were also confirmed in the other MB cell lines UW-228 and D283 (Supplementary Fig. S3).

As expected, the reduction in endogenous GLI1 protein levels following SMURF overexpression leads to a decrease in its transcriptional activity, as observed by the reduction in mRNA levels of some of its transcriptional targets (Supplementary Fig. S4).

### SMURFs expression reduces GLI1-dependent MB cell proliferation

**Fig. 6:**
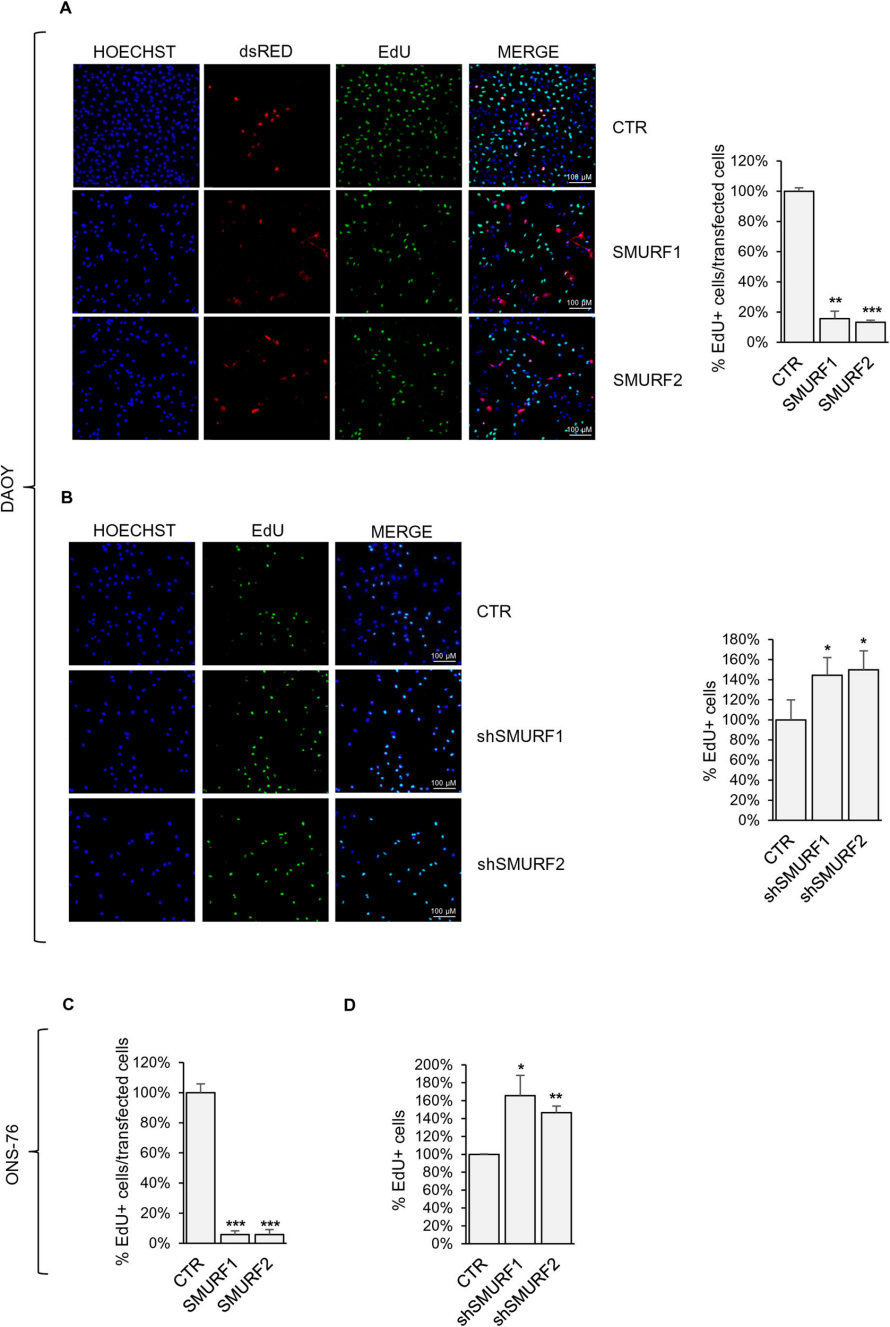
SMURFs expression reduces GLI1 dependent MB cell proliferation. EdU incorporation staining on MB cells DAOY and ONS-76 expressing SMURF proteins (**A**–**C**) or stably transduced cells with the indicated shRNAs against SMURF1 or SMURF2 (**B**–**D**). Percentage of EdU positive cells was calculated over transfected or total cells as indicated. (*p < 0.05; **p < 0.01; ***p < 0.001 results are expressed as the mean ± SD of three independent experiments, Student’s *t*-test).

After we confirmed the ability of SMURF1 and SMURF2 to regulate the ubiquitination levels of GLI1 in MB and to promote the reduction of its protein levels, we investigated whether modulating the expression of these two HECT Ubiquitin Ligases could affect the proliferation rate of MB cells. The EdU staining analysis shows that SMURF overexpression significantly reduces the proliferation rate of DAOY and ONS-76 cells, whereas their silencing increases proliferation, consistent with the increase in GLI1 protein levels (Fig. 6A–D and Supplementary Fig. S5). Similar results were observed in the UW-228 (in agreement with previously published results [20]) and D283 cell lines (Supplementary Fig. S6).

## Discussion

The Hh/GLI signaling pathway plays a crucial role in several biological processes, and its abnormal activation is associated with tumorigenesis in various types of cancers [3]. The transcription factor GLI1 is considered the main downstream effector of the Hh signaling and plays a relevant role in several cancers. In our effort to better understanding regulatory mechanisms controlling its transcriptional activity and stability, we focused on PTMs, and in particular on GLI1 ubiquitination, identifying SMURF1 and SMURF2 as modulators of GLI1 protein stability and as a consequence of the Hh signaling pathway.

SMURF proteins belong to the NEDD4 subfamily of HECT and have been identified initially as negative regulators of BMP and TGF-β signaling pathways [28]. More recently increasing evidence has suggested their involvement in modulation of the Hh pathway.

Indeed, SMURF controls PTCH1 and SMO turnover in flyes [17], consequently being able to modulate the pathway both in a positive way (by degrading PTCH1) and in a negative way (by degrading SMO). It is not clear yet, which are the signals that decide which of the two modulations will prevail in the cells. The information available so far on mammalian cells indicate a divergence in the modulatory mechanisms; in particular, in mouse fibroblasts SMURFs have been suggested to induce PTCH1 ubiquitination and degradation [18]^,^ while no observations have been published so far on the potential modulation of SMO. Of interest, SMURF-driven degradation of PTCH1 seems to be mediated by lysosomal degradation [18].

More recently, Li and colleagues have reported that SMURFs may act on the Hh pathway through an epigenetic mechanism driven by degradation of RNF220 [20]. RNF220 is a RING Finger protein which increases gene expression of target genes (including GLI1 itself) by inducing degradation of EED, a component of the transcription repressor PRC2 [19].

Our data, presented here, confirm the negative role of SMURFs in the modulation of the Hh/GLI pathway, but we highlight a different and more direct mechanism through which SMURFs can modulate the Hh pathway.

We demonstrate that SMURF1 and SMURF2 can act directly on GLI1 protein, revealing a new level of SMURF modulation on the Hh pathway.

In fact, we proved that SMURFs interact directly with proline-rich regions within the a.a. 424-1106 of GLI1, and through this interaction can recruit the ubiquitination machinery and promote GLI1 polyubiquitination.

Of interest, the ubiquitination induced by SMURFs on GLI1 is not the K63-polyubiquitination (that drives lysosomal degradation) but is the K48-polyubiquitination which leads to GLI1 proteasomal degradation [22]. Overexpression of SMURFs, reducing GLI1 levels, induces Hh pathway downregulation and has a significant effect on Hh-driven cell proliferation and in particular in Hh-dependent cancer cells proliferation. Indeed, we demonstrated that SMURFs overexpression in MB cancer cell lines reduces cell proliferation significantly. The biological effect of SMURFs overexpression presented here agrees with previous findings which demonstrated that Hh/GLI suppression induces a significant reduction in cancer cell proliferation, both in vitro and in vivo [20, 29, 30].

Interestingly, in silico analysis on existing databases of MB human samples highlights a significant inverse correlation between *Smurfs* gene expression and *Gli1* (Supplementary Fig. S7).

The data from this and previous studies suggest multiple roles for SMURFs on modulation of the Hh pathway, including a positive modulation through the degradation of PTCH1 and two negative modulations: at the level of repression of gene transcription or directly through modulation of GLI1. It remains to be clarified if the two SMURFs proteins perform the same identical task or if they have physiologically different propensity for the different targets. Also, the two SMURFs may be expressed at different levels and at different times during and their functional redundancy is essential to grant a continuous control of the pathway. Why positive and negative effect are attributed to the same molecules is also an interesting issue: it is possible that the positive or negative modulation of the Hh pathway, and the use of a direct or indirect mechanism of action on GLI1 stability or transcriptional activity may depend on the different cellular and developmental contexts.

Without any doubt, this fine, tight and redundant mechanism of regulation underlines how critical it is for the cellular and tissue homeostasis, the presence of SMURFs proteins.

Indeed, the two HECT-E3 ligases demonstrate the ability to regulate Hh signaling pathway at multiple levels, with diametrically opposed effects. By regulating the turnover of PTCH1, they promote pathway activation following the binding between Hh and its receptor. It is likely that this positive signal is more relevant during development and in context driven by Hh binding to PTCH1. In contexts requiring pathway attenuation, SMURF1 and SMURF2 act as negative regulators by downregulating GLI1 protein levels or its transcriptional activity.

Considering that a significant part of the Hh-dependent tumors is not driven exclusively by the Hh ligand, but most often they present also a non-canonical activation which acts at the level of GLI, the negative modulatory role of SMURFs seems to have a prevalent effect on tumor cell proliferation.

Of note, we have also verified that SMURF negative modulation on GLI1 is present, as expected, also in a PTCH1-KO context (*Ptch1*-KO mouse embryonic fibroblasts; Supplementary Fig. S8).

Overall, our results demonstrated a novel modulatory role of SMURF1 and SMURF2 on GLI1, elucidating the molecular mechanisms underlying this regulation.

The observation of a negative correlation between *Smurf* genes expression and Hh activation in MB tumors suggests that the mechanism of Hh modulation driven by SMURFs plays an important role either in the tumorigenetic process or in the progression of Hh driven tumors. Previous observations on MB cells [20], and our data, suggest that restoring SMURF expression in the tumoral context may be a novel promising approach for MB treatment, and potentially for the treatment of other tumors involving aberrant Hh-GLI activation, especially if non canonically activated.

## Material and methods

### Cell lines, transfection and treatment

HEK-293T, MEF PTCH1-KO, NIH3T3, UW-228 cell lines were cultured in Dulbecco’s Modified Eagle Medium (DMEM) (Sigma-Aldrich, St. Louis, United States), in a humidified incubator at 37 °C with 5% CO_2_ supplemented with 10% FBS, 1% Penicillin and Streptomycin, and 1% Glutamine. Mycoplasma contamination in cell cultures was routinely assessed using a PCR detection kit (Applied Biological Materials, Richmond, BC, Canada).

The DAOY medulloblastoma cell line was cultured in Minimal Essential Medium (MEM) (Sigma-Aldrich, St. Louis, United States), supplemented with 10% FBS, 1% Sodium Pyruvate, 1% Non-Essential Amino Acids, 1% Glutamine and 1% Penicillin and Streptomycin.

The ONS-76 medulloblastoma cell line was cultured in RPMI-1640 medium (Sigma-Aldrich, St. Louis, United States), supplemented with 10% FBS, 1% Glutamine and 1% Penicillin and Streptomycin.

The MB-D283 medulloblastoma cell line was cultured in Minimal Essential Medium (MEM) (Sigma-Aldrich, St. Louis, United States), supplemented with 20% FBS, 1% Sodium Pyruvate, 1% Non-Essential Amino Acids, 1% Glutamine and 1% Penicillin and Streptomycin.

Transfection of HEK-293T cells was performed using the DreamFectTM Gold transfection reagent (OZ BIOSCIENCES). The MEF PTCH1-KO, DAOY, ONS-76, D283, UW-228 cells were transfected with Lipofectamine 2000 (Invitrogen). All transfections were conducted following the manufacturers’ instructions.

HEK-293T were treated with 1 µM MG132, a proteasomal inhibitor, for 16 h at 37 °C in complete DMEM. Dimethyl sulfoxide (DMSO) (Sigma) was added in equivalent amounts as a control.

### Lentivirus infection and cell line construction

The following shRNA sequences targeting human SMURF1 and SMURF2 were synthesized and cloned into the lentiviral knockdown vector pLKO.1: shSMURF1: 5′-GCCCAGAGATACGAAAGAGAT-3′, and ShSMURF2: 5′-CCACCCTATGAAAGCTATGAA-3′. For lentiviral infection, HEK-293T cells were transfected with pLKO.1 plasmids shCTR ShC002, ShSMURF1 or shSMURF2, and the packaging plasmids pCMV-dR8.74 and VSV-G/pMD2 using Calcium/ Phosphate precipitation. Culture medium containing the lentivirus was collected 48 and 72 h after transfection. DAOY, ONS-76 and UW-228 were infected with purified lentivirus in presence of polybrene (Sigma Aldrich, St Louis, Mo) and the stably transfected cells were selected with puromycin for 72 h.

### Plasmids

The plasmids pCDNA-GLI1-3xFLAG, pCDNA-GLI1-3XHA, pCDNA-GLI1(424-1106)-3xFLAG, pCDNA-GLI1(1-413)-3xFLAG and pCDNA-GLI1(TM)-3xFLAG have been generated in our laboratory. The vector pCDNA 3.1 was obtained from Invitrogen.

The vectors pCMV5B-FLAG-SMURF1, pRK1-MYC-SMURF1, pRK1-MYC-SMURF2, pCDNA-HA-Ubiquitin, pCMV-MYC-Ubiquitin were purchased from Addgene. The GLI1-12xLuc and pRL-TK-Renilla reporter vectors were kindly supplied by R. Toftgard (Karolinska Institutet, Stockholm).

### Luciferase assay

The luciferase assay on the HEK-293T cell line was conducted using the Firefly Luciferase Assay 2.0 kit (Biotium, California, United States), following the manufacturer’s instructions as previously described [31]. Luminescence was measured using the GloMax®R Discover Microplate Reader (Promega).

### RNA extraction, cDNA synthesis and quantitative real-time PCR

Total RNA extraction from cells was performed using the TRIzol reagent (Invitrogen Life Technologies), according to the manufacturer’s instructions. Subsequently, the RNA Clean and ConcentratorTM-5 kit (R1014, Zymo Research, California, United States) was used for RNA purification. 1 μg of RNA was reverse transcribed using the high-capacity cDNA reverse transcription kit (BIO-65054, Meridian Bioscience, OH, United States). Expression level of target genes was quantified using TaqMan^TM^ Gene Expression Assay (Applied Biosystem-Thermo Fisher Scientific) or SYBR Green qPCR Master Mix (Thermo Fisher Scientific), on a ViiATM7 Real-Time PCR system (Applied Biosystem-Thermo Fisher Scientific). All reactions were run at least in triplicate.

We used the following TaqMan^TM^ assays: GLI1 (Hs01110766_m1), VEGF-A (Hs00900055_m1), Cyclin D2 (Hs00153380_m1) and PTCH1 (Hs00181117_m1). RNA levels were normalized to the average expression of housekeeping genes such as β2M (4326319E), TBP (4326322E), GAPDH (4310884E) or β-Actin (4333762E), to minimize the effect due to potential fluctuations of single housekeeping [32].

The following primers were used for SYBR Green qPCR:

hGLI1 Fw: 5′-GGGATGATCCACATCCTCAGCT-3′;

hGLI1 Rv: 5′- CTGGAGCAGCCCCCCCAGT-3′;

hCyclin D2 Fw: 5′-GCAGAAGGACATCCAACCCTAC-3′;

hCyclin D2 Rv: 5′- TGGCCAGAGGGAAGACCTCT-3′;

hβ-Actin Fw: 5′-CACCCTGAAGTACCCCATCGAG-3′;

hβ-Actin Rv: 5′- TGATCTGGGTCATCTTCTCGCG-3′;

hGAPDH Fw: 5′- AACAGCGACACCCATCCTC-3′;

hGAPDH Rv: 5′- CATACCAGGAAATGAGCTTGACAA-3′.

### Immunoblotting

Transfected cells were lysed using SDS-urea buffer (50 mM Tris HCL pH 7.8; 10% Glycerol; 2% SDS; 10 mM EDTA pH 8.0; 100 mM NaF; 10 mM Na2P2O7; 6 M Urea) or RIPA lysis buffer (Tris-HCl 50 mM, NaCl 150 mM, Triton X-100 1%, SDS 0.1%, DOC 0.5%, EDTA 1 mM) supplemented with protease inhibitors (10 μg/ml aprotinin, 10 μg/ml pepstatin, 10 μg/ml leupeptin, 1 mM PMSF, 2 mM Na3VO4).

Total protein extracts were then evaluated by SDS-PAGE using the antibodies listed below: mouse monoclonal anti-FLAG M2 HRP (F3165; Sigma-Aldrich), mouse monoclonal anti-HA (SC-7392; Santa Cruz Biotechnology), rabbit monoclonal anti-c-MYC (C3966; Sigma-Aldrich Merck), rabbit polyclonal anti-SMURF1 (#2174; Cell Signaling), rabbit monoclonal anti-SMURF2 (#12024; Cell Signaling), rabbit polyclonal anti-GLI1(H-300) (SC-20687; Santa Cruz Biotechnology), mouse monoclonal anti-β-ACTIN (C4) HRP (sc-47778; Santa Cruz Biotechnology), mouse monoclonal anti-VINCULIN (7F9) HRP (sc-73614; Santa Cruz Biotechnology). Secondary antibody anti-mouse (SC-516102; Santa Cruz Biotechnology) or anti-rabbit (SC-2357; Santa Cruz Biotechnology) conjugated with HRP. Immunocomplex were detected using a chemiluminescence reaction. Detection of the HRP signal was performed by using ECL (#K-12045-D50, Advansta).

### Co-immunoprecipitation

Cells were lysed with Triton X-100-containing lysis buffer (25 mM HEPES, 100 mM NaCl, 1 mM EDTA, 10% Glycerol, 1% Triton X-100), and protease inhibitors (10 μg/ml aprotinin, 10 μg/ml pepstatin, 10 μg/ml leupeptin, 1 mM PMSF, 2 mM Na3VO4) for 30 min on ice and then centrifuged at 13,000 rpm for 30 min at 4 °C. The supernatants were then incubated for 2 h with anti-FLAG conjugated (A2220; Sigma Aldrich-Merk)/anti-HA conjugated (A2095; Sigma Aldrich-Merck) agarose beads or with a primary anti-GLI1 (H-300) antibody (SC-20687; Santa Cruz Biotechnology) followed by incubation with Protein A agarose beads (Sc-2001; Santa Cruz Biotechnology) for 2 h on a rotating wheel at +4 °C. As a negative control, the agarose beads were saturated with the FLAG peptide (F3290; Sigma Aldrich-Merk)/ HA peptide (26184; Thermo Fisher Scientific) or IgG (A82271). The beads were then washed five times with a Washing Buffer (50 mM Tris HCL pH 7.6; 150 mM NaCl; 0.5% NP-40; 5 mM EDTA pH 8; 100 mM NaF). Finally, the protein complexes were analysed via SDS-PAGE.

The in vitro co-immunoprecipitation assays were performed using the Cell-free protein expression kit (TnT® Coupled Reticulocyte Lysate Systems, Promega). The reaction mix contains TNT Rabbit Reticulocyte, TNT Reaction Buffer, TNT RNA polymerase, Transcent Biotin-Lysyl-tRNA, amino acid minus methionine, Methionine 1 mM, RnasiOUT, and pCDNA-GLI1(424-1106)-3xFLAG, pCDNA-GLI1(1-413)-3xFLAG, pRK1-MYC-SMURF1, pRK1-MYC-SMURF2. GLI1 and SMURFs in vitro translated proteins expressed were incubated together for 2 h on a rotating wheel at +4 °C and then co-immunoprecipitated as previously described.

### Ubiquitination assay

Cells were lysed in Triton X-100-containing lysis buffer as previously described and then immunoprecipitated by anti-FLAG agarose beads, anti-HA resin or with anti-GLI1 (H-300) antibody. Ubiquitin residues were detected using anti-c-MYC (C3966; Sigma-Aldrich Merck), anti-FLAG M2 HRP (F3165; Sigma-Aldrich), anti-K48-linkage Specific Polyubiquitin Antibody (#4289; Cell Signaling) and anti-K63-linkage Specific Polyubiquitin Antibody (#5621; Cell Signaling)

The in vitro ubiquitination assays were performed in a 50 μL reaction volume containing the following components: HA-Ubiquitin Protein (U-110-1MG; R&D Systems); Ubiquitin-activating enzyme E1 (E-304-050; R&D Systems); Ubiquitin-conjugating enzyme E2 UbcH5a (E2-616-100; R&D Systems); E3 Ubiquitin-ligases SMURF1 or SMURF2 (from in vitro translated proteins production); and GLI1(1-413)-3xFLAG or GLI1(424-1106)-3xFLAG (immunoprecipitated with anti-FLAG agarose beads from in vitro translated proteins production); 10X reaction buffer and Mg-ATP Solution (B-20; R&D Systems). Reactions were incubated at 37 °C for 2 h and then washed five times with a Washing Buffer. The proteins were eluted and then analysed by SDS-PAGE.

### EdU proliferation assay

The EdU proliferation assay on the DAOY, ONS-76, D283 and UW-228 cell lines was conducted using the Click-iT™ EdU Cell Proliferation Kit for Imaging (C10337; Invitrogen), following the manufacturer’s instructions as previously described [33]. Identification of transfected cells was performed transfecting, together with the plasmids coding for the gene of interest, a plasmid coding for DSRed fluorescence protein (1/10 of the total amount of DNA).

### Proximity ligation assay (PLA)

HEK-293T and NIH3T3 cell lines were cultured in an 8-well chamber slide and HEK-293T were transfected for 24 h with pCDNA-GLI1-3xFLAG.

The cells were then fixed for 30 min at room temperature with 4% paraformaldehyde, before permeabilization and incubation with primary antibodies. Primary antibodies which were used are: mouse monoclonal anti-SMURF1 (sc-100616; Santa Cruz Biotechnology), rabbit monoclonal anti-SMURF2 (#12024; Cell Signaling), mouse monoclonal anti-GLI1 (sc-515781; Santa Cruz Biotechnology) or rabbit monoclonal anti-FLAG (F7425-2MG; Sigma) diluted in blocking solution. As a negative control the cultures were processed in absence of primary antibody. The subsequent steps of the assay were performed according to the manufacturer’s instructions (DUO92101; Sigma).

### Public dataset gene expression analysis

R2-Genomics analysis and visualization platform (http://r2.amc.nl) were used for gene expression analysis.

### Statistical analysis

For all luciferase, qPCR and EdU Proliferation Assay, the *p* values were determined using Student’s *t*-test and statistical significance was set at *p < 0.05, **p < 0.01 or ***p < 0.001. Results are expressed as mean ± S.D. All experiments were replicated biologically at least three times.

## Supplementary information

supplementary figures

Full scan of all western blot

## Data Availability

All data included in this study are available upon request by contact with the corresponding author.
