# Supplementary material for: SMURF1 and SMURF2 directly target GLI1 for ubiquitination and proteasome-dependent degradation
Source: Cell Death Discov. 2024 Dec 18;10:498. doi: 10.1038/s41420-024-02260-4 (PMC11655642; doi:10.1038/s41420-024-02260-4)
Supplement: Supplementary file 1 — supplementary figures [file 41420_2024_2260_MOESM1_ESM.pdf]

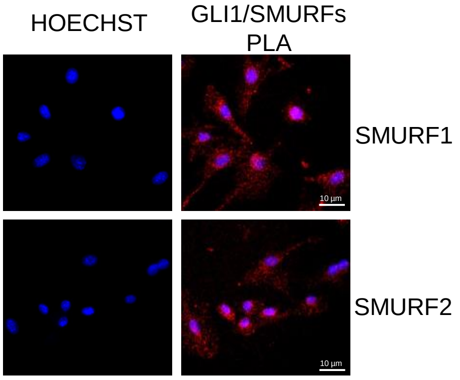

Supplementary Figure S1. PLA assay detects protein interaction between endogenous GLI1 and SMURFs in Hh-responsive NIH3T3 cells.

Proximity ligation assay (PLA) for endogenous SMURFs and GLI1 interaction. NIH3T3 cells were fixed in paraformaldehyde (PFA) and permeabilized. Subsequently, cultures were processed with primary antibody anti-GLI1 and either anti-SMURF1 or anti-SMURF2, and with specific secondary antibodies for PLA assay (red signal), as described in Methods. Nuclei were stained blue (Hoechst).

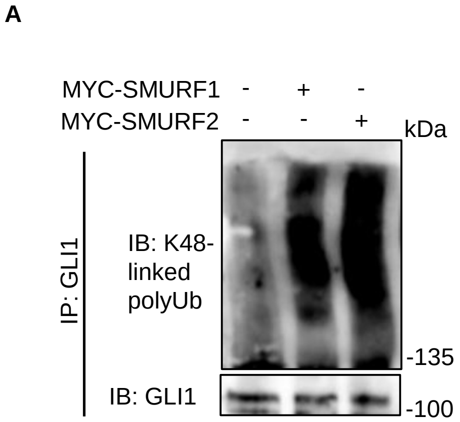

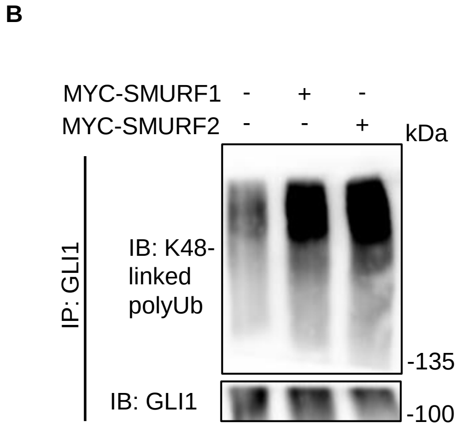

Supplementary Figure S2. SMURFs expression triggers degradative K48-linked polyubiquitination of GLI1.

A-B. Ubiquitination assays on DAOY (**panel A**) and ONS-76 (**panel B**) cells expressing either control vector, MYC-SMURF1 or MYC-SMURF2. 24h after transfection, cells were lysed, and the ubiquitination status was evaluated after endogenous GLI1 immunoprecipitation. Proteins were detected using antibodies anti-GLI1 and anti-K48-linkage specific polyubiquitin

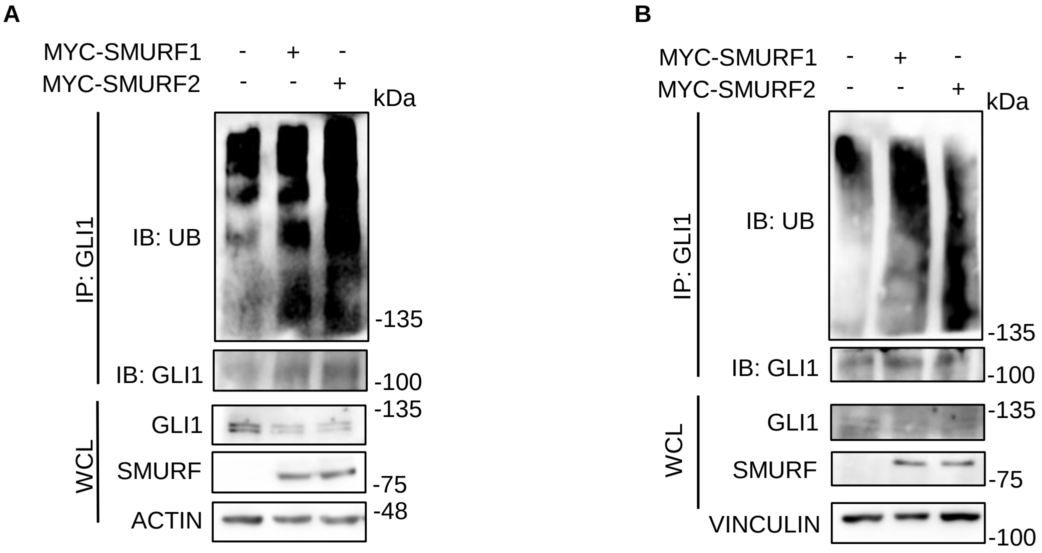

Supplementary Figure S3. SMURFs expression promotes GLI1 ubiquitination and degradation in D283 and UW-228 MB cell.

A-B. Ubiquitination assays on D283 and UW-228 cells expressing either control vector, MYC-SMURF1 or MYC-SMURF2. 24h after transfection, cells were lysed, and the ubiquitination status was evaluated following endogenous GLI1 immunoprecipitation. Proteins were detected using antibodies anti-GLI1, anti-SMURF1, anti-SMURF2, anti-Ubiquitin and anti-ACTIN (used as a normalizer).

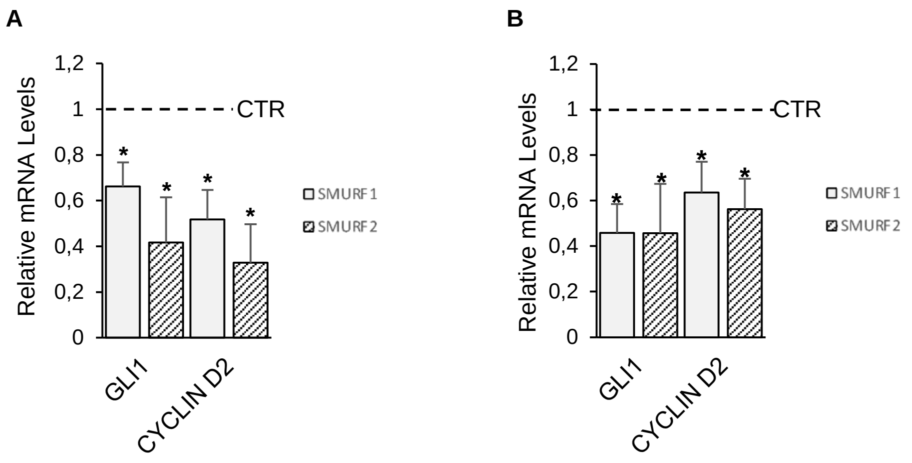

Supplementary Figure S4. SMURFs overexpression reduces GLI1 targets expression in D283 and DAOY MB cells.

A-B. RT-qPCR analysis of Hh/GLI1 target genes expression. D283 (**panel A**) and DAOY (**panel B**) cells were lysed for mRNA extraction 24h after transfection with MYC-SMURF1 or MYC-SMURF2. mRNA levels were analysed by RT-qPCR. Gli1 and Cyclin D2 mRNA expression was normalized to the average expression of two housekeeping genes.

A

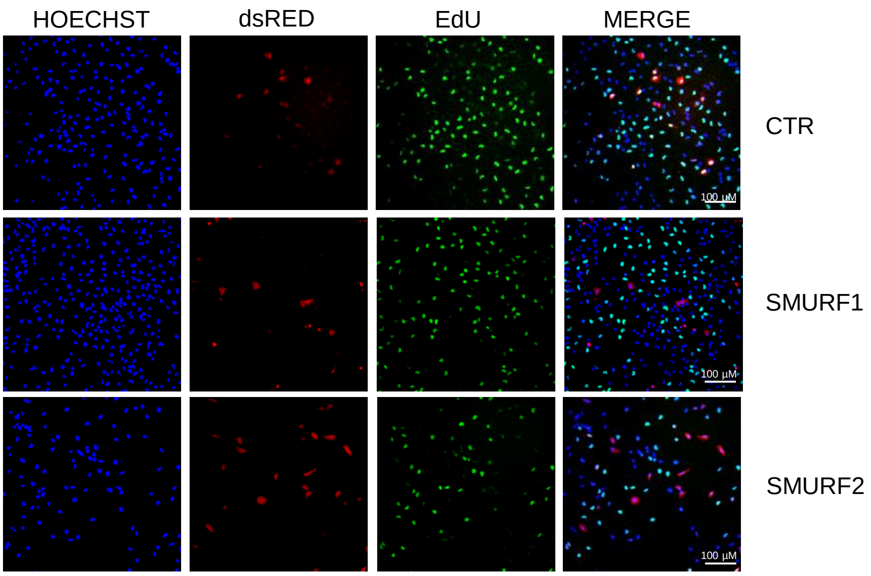

B

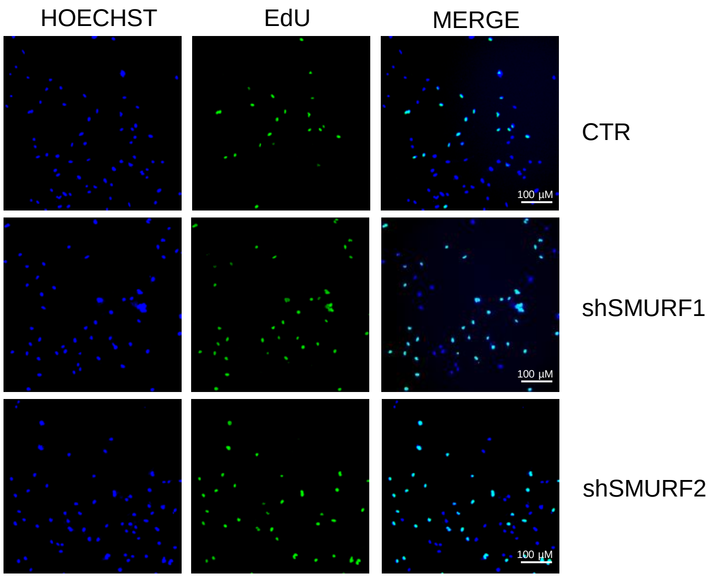

Supplementary Figure S5. SMURFs expression reduces GLI1 dependent ONS-76 MB cell proliferation.

A-B. EdU incorporation assay on ONS-76 cells expressing SMURF proteins (**panel A**) or stably transduced with shRNAs against SMURF1 or SMURF2 (**panel B**).

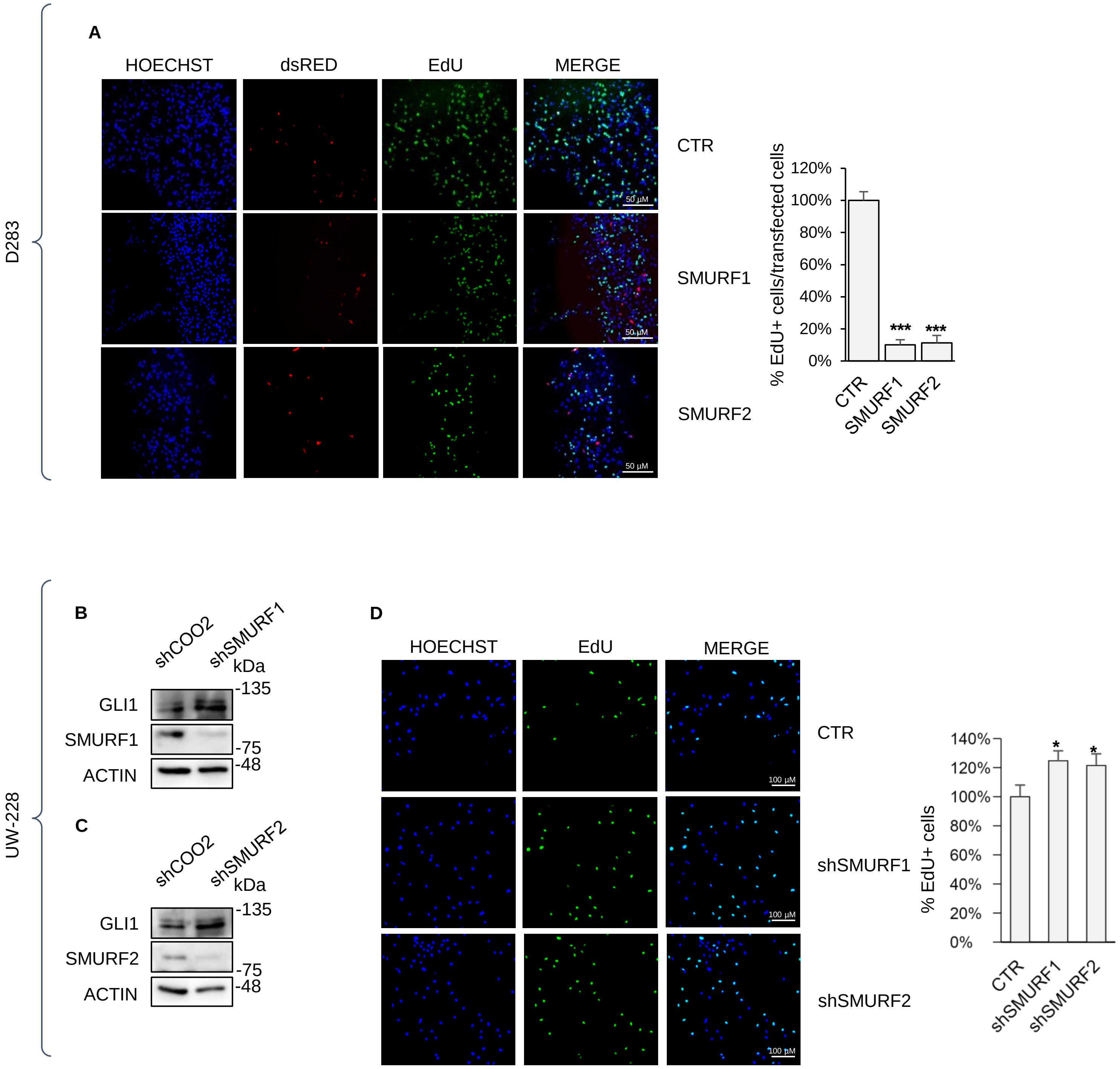

Supplementary Figure S6. SMURFs modulation affects D283 and UW-228 MB cell proliferation by increasing or decreasing Gli1 protein levels.

A. EdU incorporation staining on D283 cells expressing either SMURF1 or SMURF2. Percentage of EdU positive cells was calculated over total cells. (*$p < 0.05$; results are expressed as the mean ± SD of three independent experiments, Student's t-test). **B-C.** Analysis of GLI1 protein levels following SMURFs silencing. UW-228 cells were stably transduced with shRNAs against SMURF1 or SMURF2. Cell lysates were analysed using SDS-PAGE and antibodies anti-GLI1, anti-SMURF1, anti-SMURF2 and anti-ACTIN (used as a normalizer). **D.** EdU incorporation assay on UW-228 MB cells stably transduced with shRNAs against SMURF1 or SMURF2. Percentage of EdU positive cells was calculated over transfected or total cells as indicated. (*$p < 0.05$; **$p < 0.01$; ***$p < 0.001$, results are expressed as the mean ± SD of three independent experiments, Student's t-test).

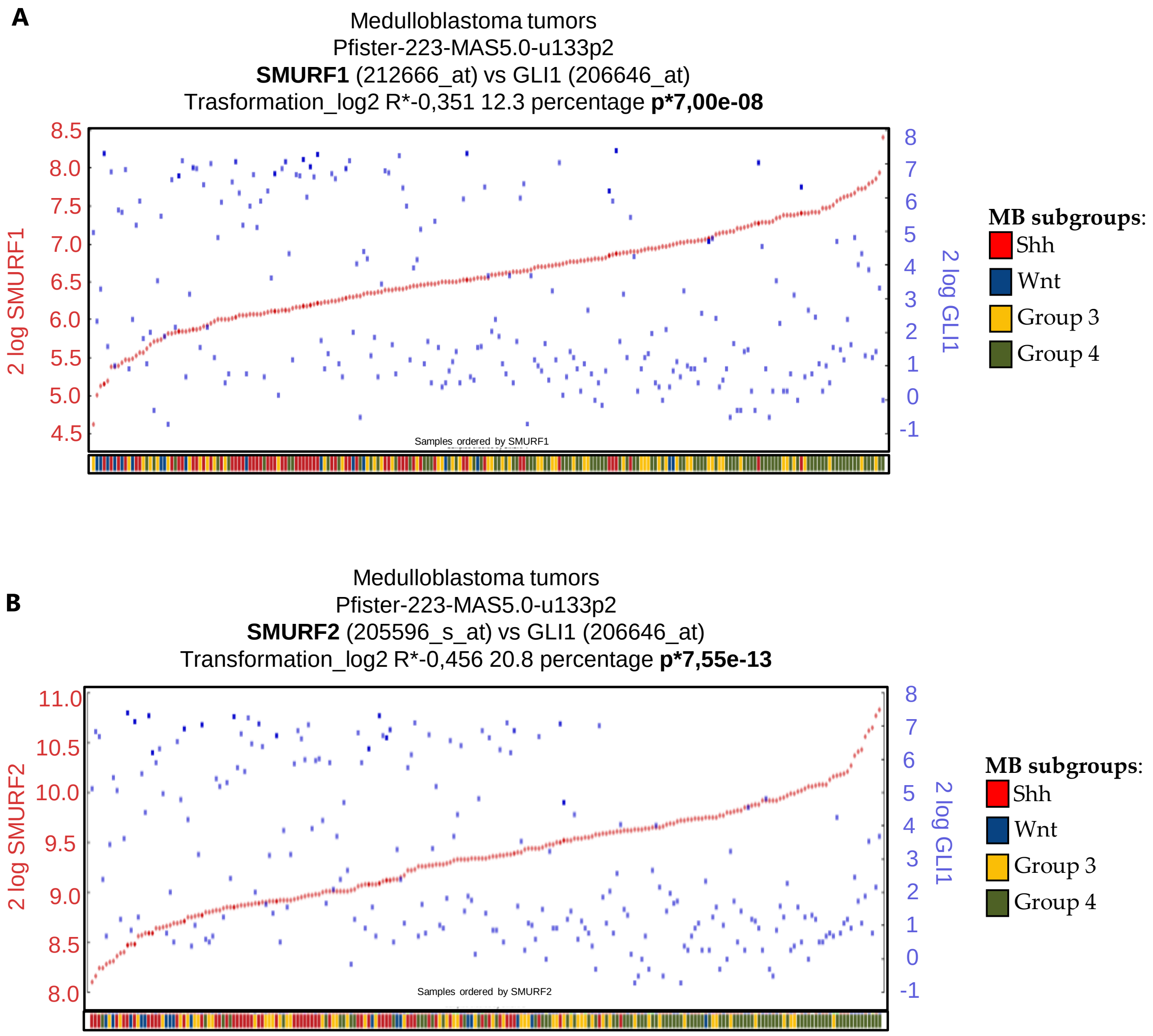

Supplementary Figure S7. Inverse correlation between Gli1 and Smurf1/2 expression in human MB samples.

A-B. In silico analysis conducted using the R2 genomic analysis and visualization platform. The correlation plot represents Gli1 and Smurf1/Smurf2 expression levels of a cohort of 223 MB patients from all 4 molecular subgroups (Northcott PA, Buchhalter I, Morrissy AS, Hovestadt V, Weischenfeldt J, Ehrenberger T et al. The whole-genome landscape of medulloblastoma subtypes. Nature. 2017 Jul 19;547(7663):311-317.).

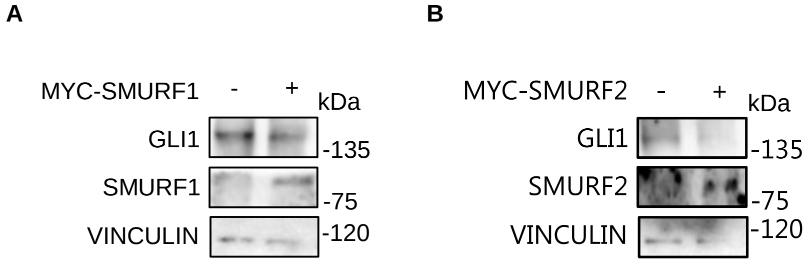

Supplementary Figure S8. SMURF1 and SMURF2 overexpression triggers GLI1 endogenous protein decrease also in a PTCH1-KO context.

A-B. Analysis of SMURFs overexpression effects on GLI1 protein levels. PTCH1-KO MEF cells were transfected with either SMURF1 or SMURF2 coding vectors. 24h after transfection, cells were lysed, and proteins were analysed by SDS-PAGE. Proteins have been detected using antibodies anti-GLI1, anti-MYC and anti-VINCULIN (used as a normalizer).
