# Supplementary material for: SMURF1 and SMURF2 directly target GLI1 for ubiquitination and proteasome-dependent degradation
Source: Cell Death Discov. 2024 Dec 18;10:498. doi: 10.1038/s41420-024-02260-4 (PMC11655642; doi:10.1038/s41420-024-02260-4)

FIGURE 1A

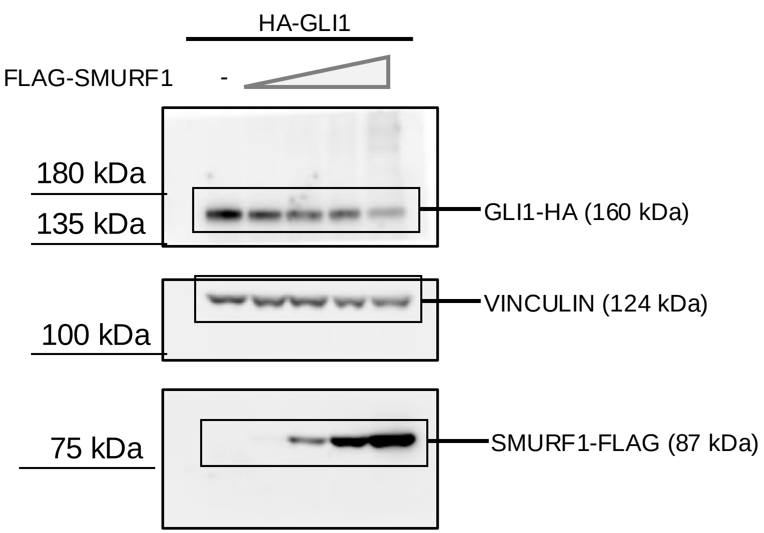

FIGURE 1B

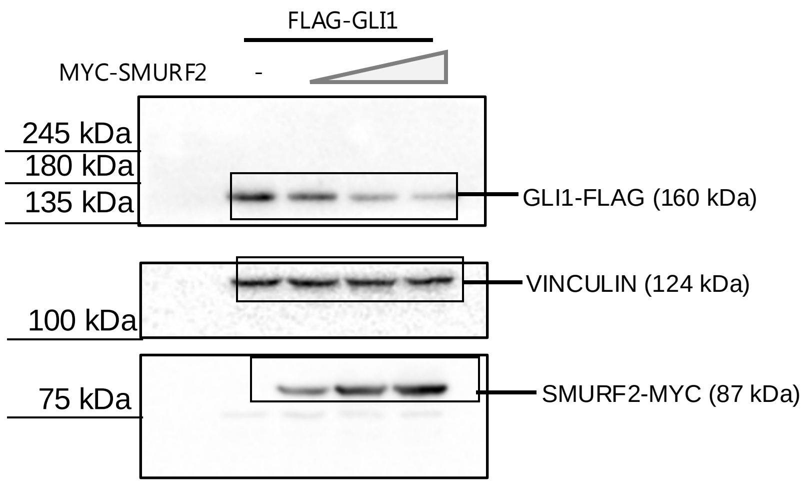

FIGURE 1C

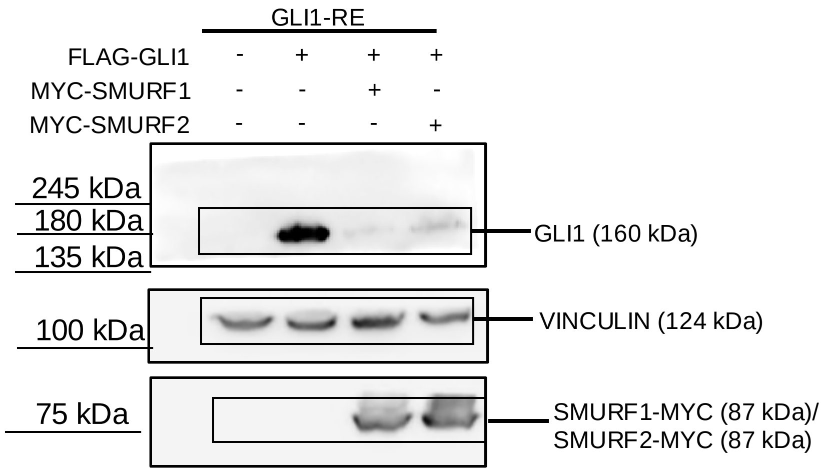

FIGURE 1D

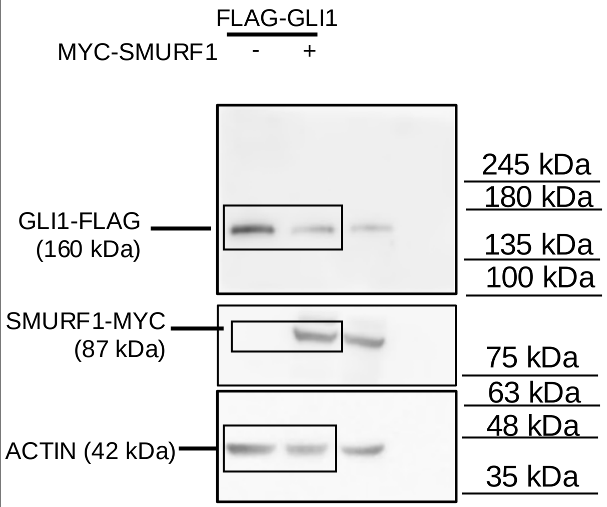

FIGURE 1E

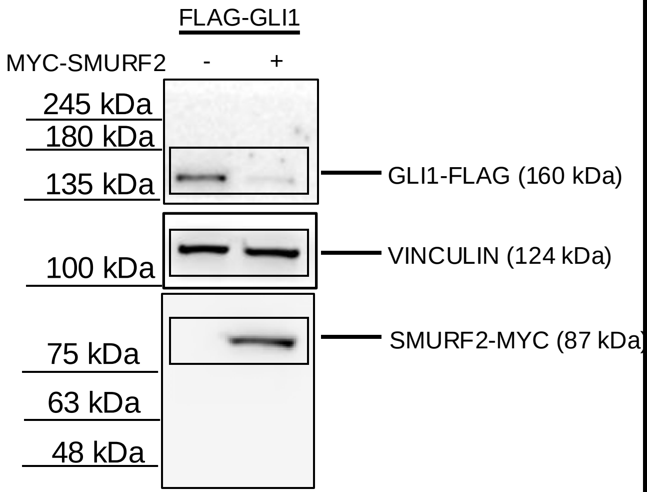

FIGURE 1G

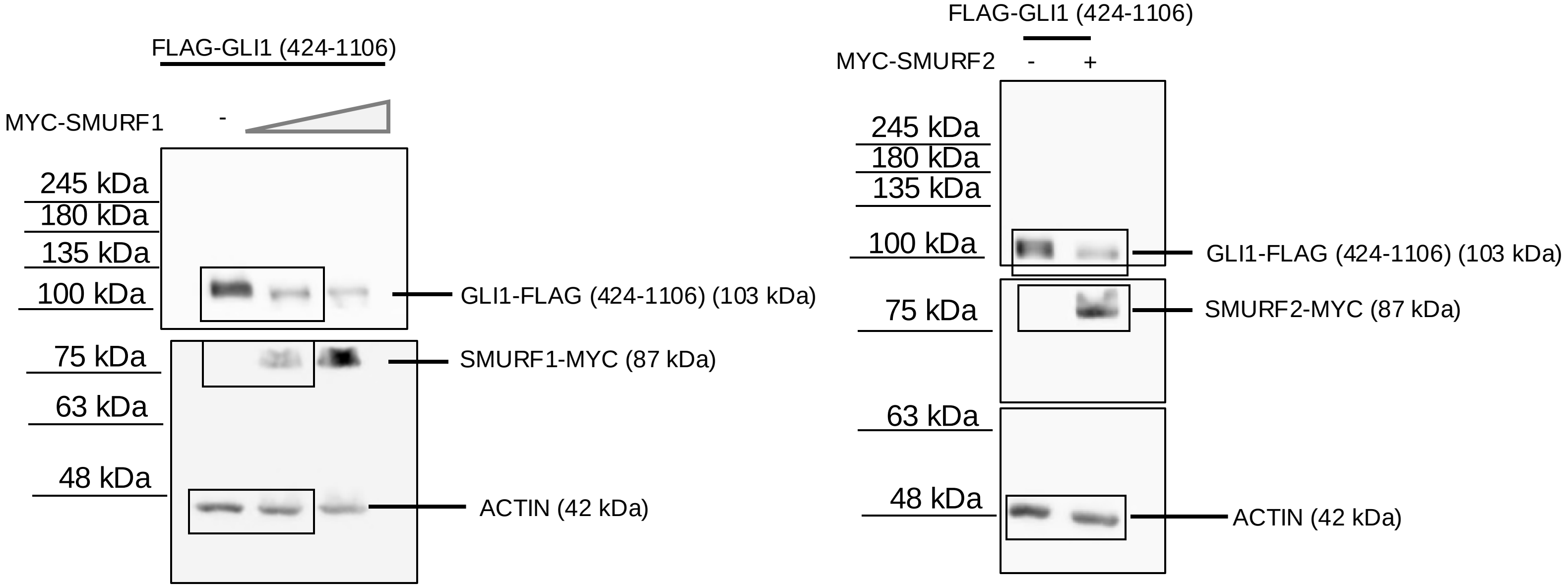

FIGURE 1H

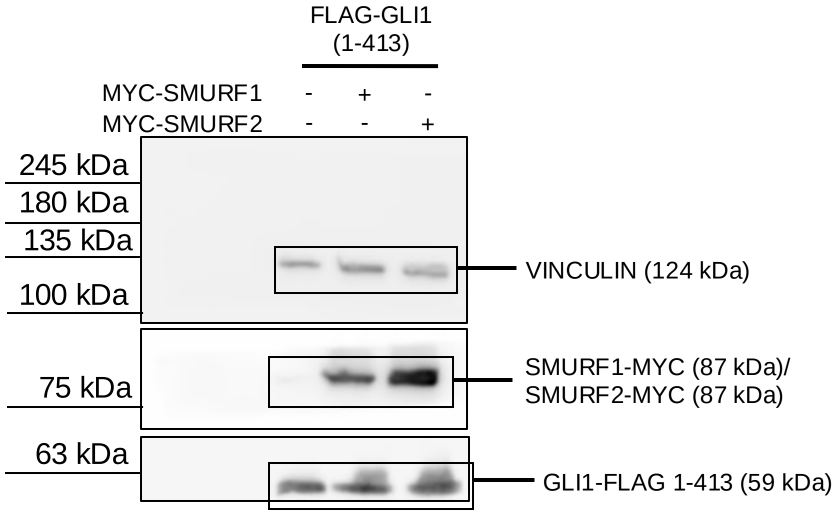

FIGURE 2A

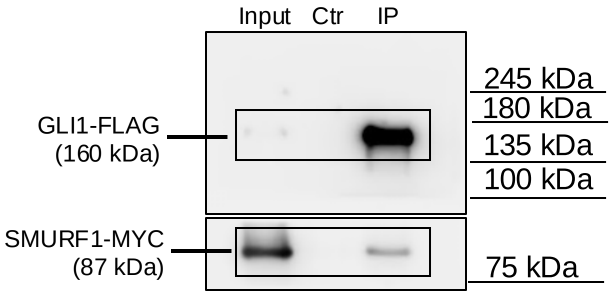

FIGURE 2B

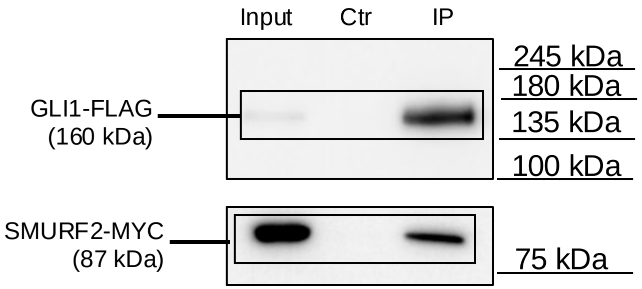

FIGURE 2D

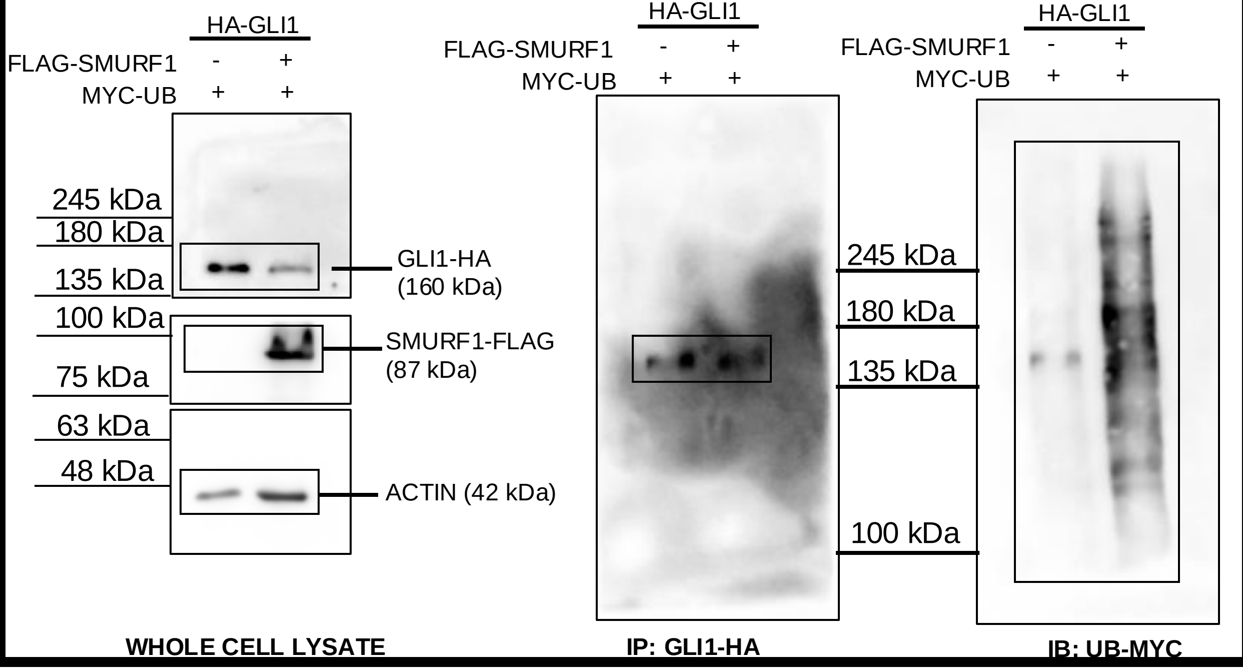

FIGURE 2E

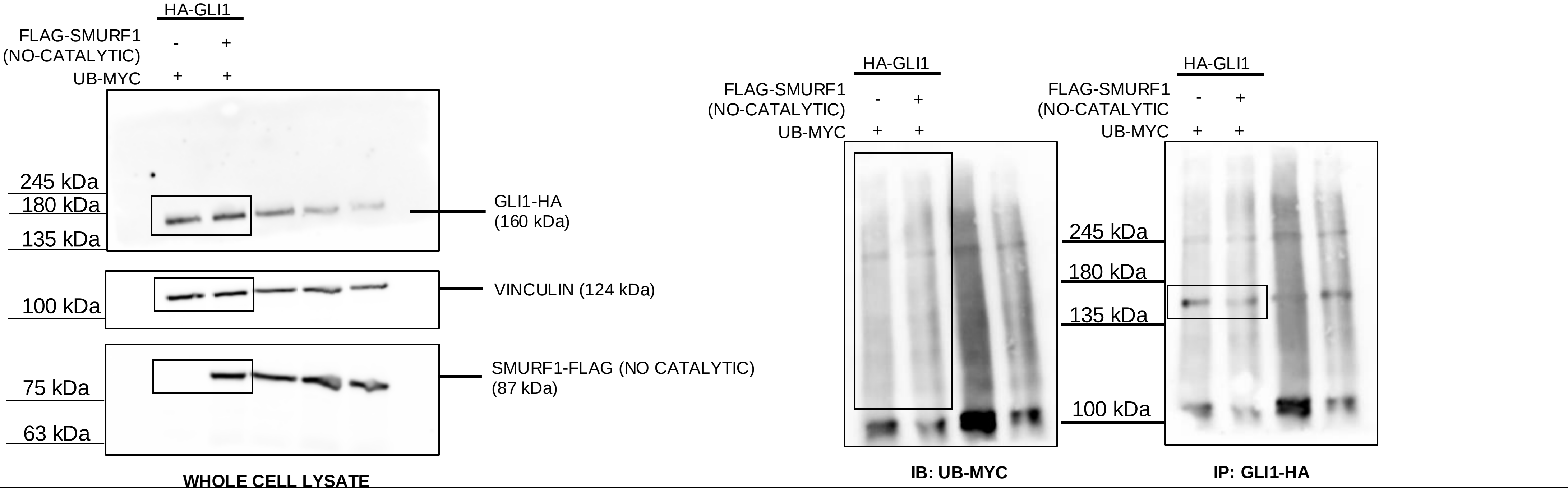

FIGURE 2F

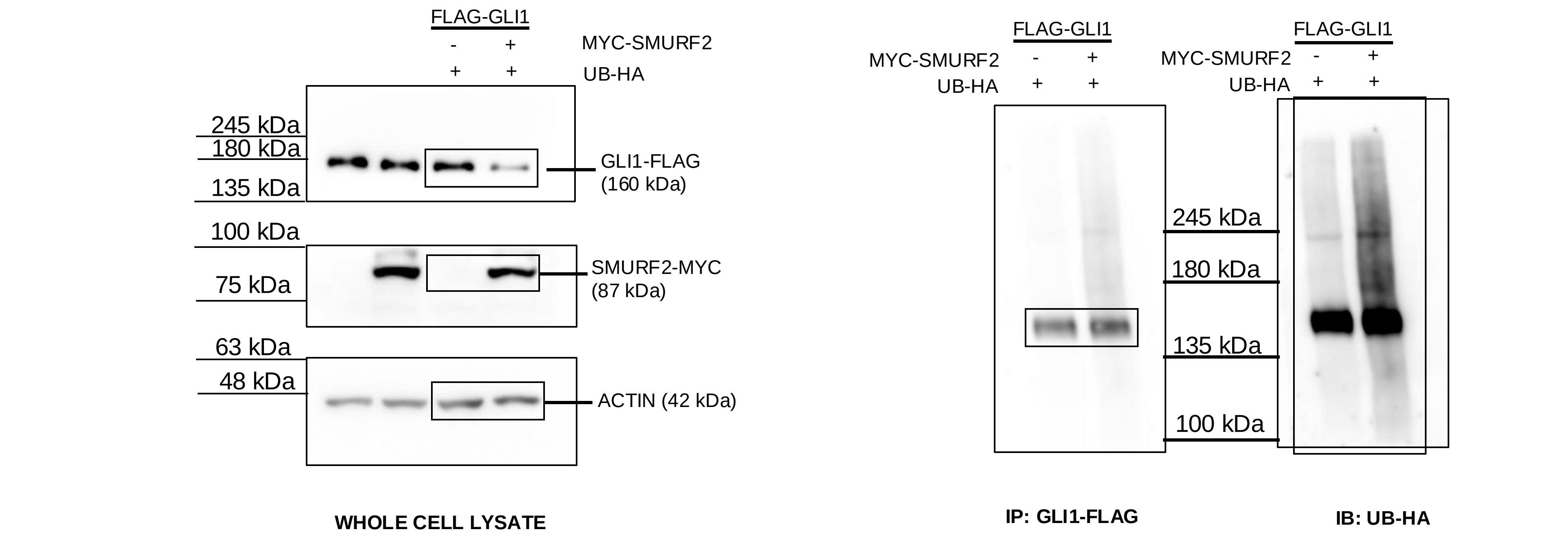

FIGURE 2G

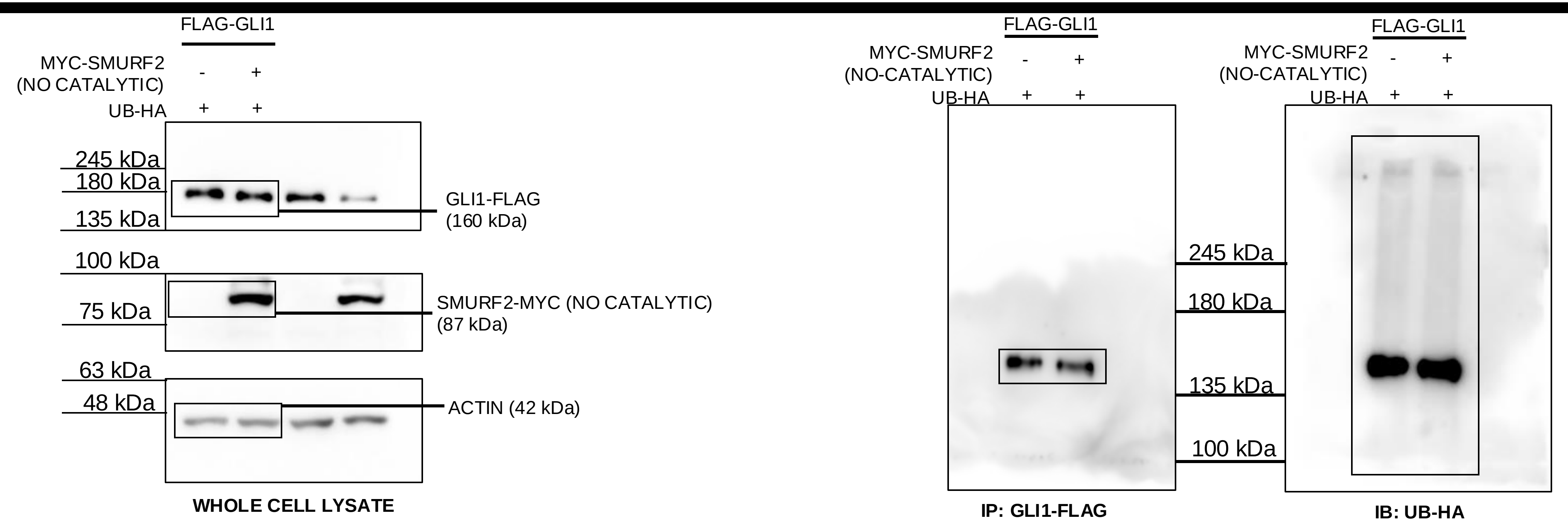

FIGURE 3A

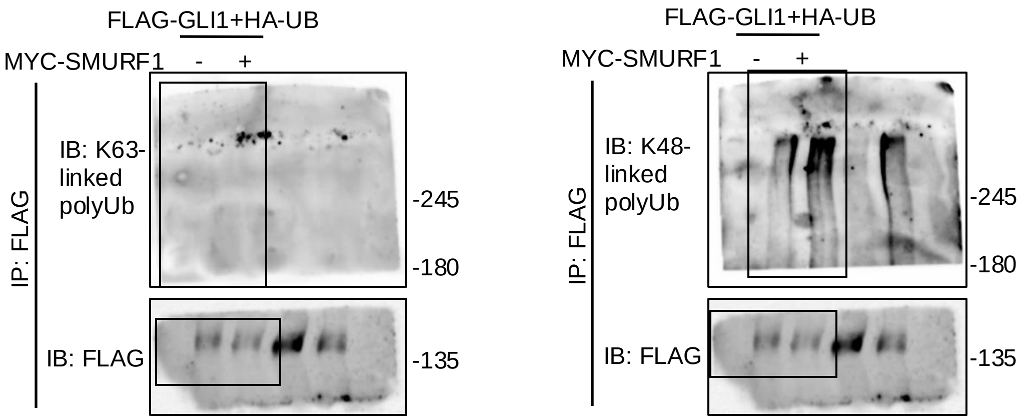

FIGURE 3B

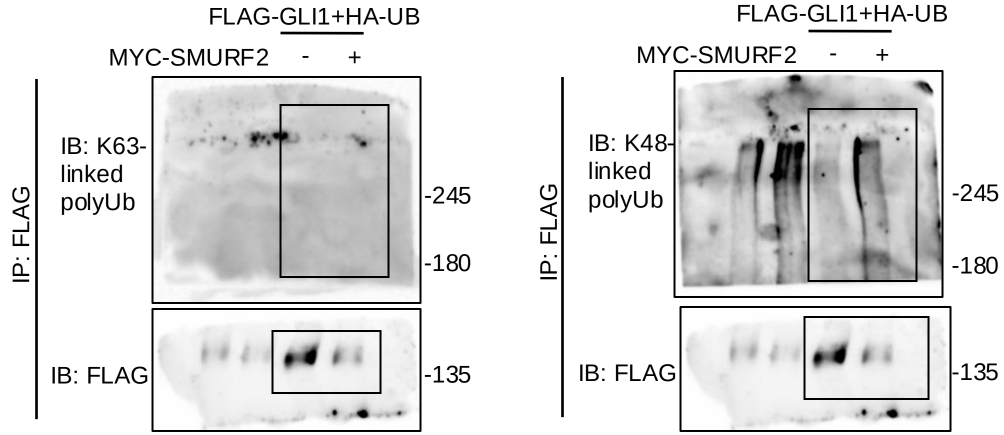

FIGURE 3C

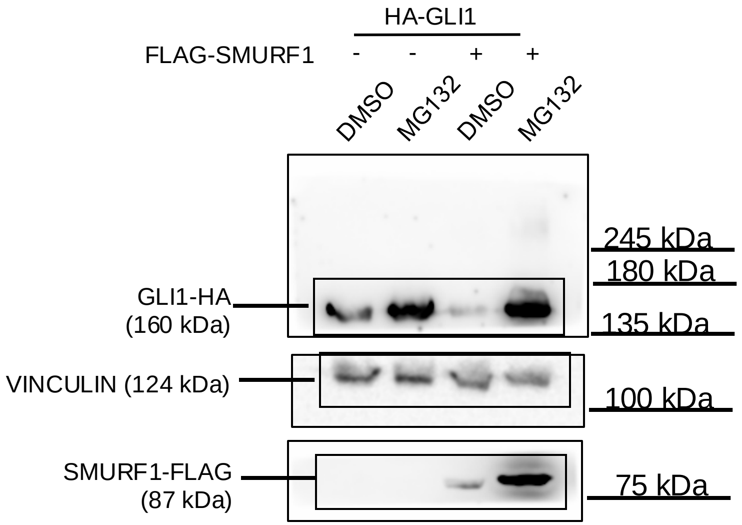

FIGURE 3D

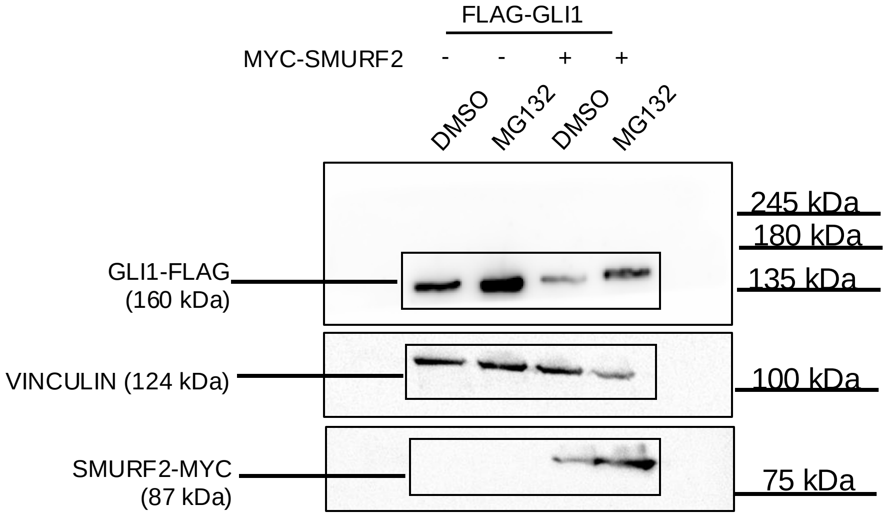

FIGURE 4A

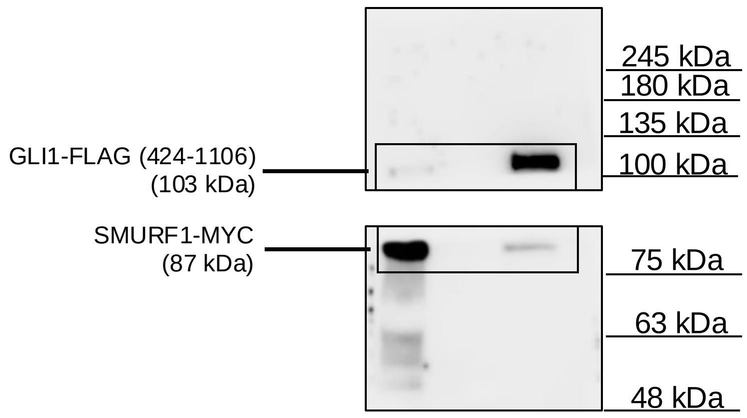

FIGURE 4B

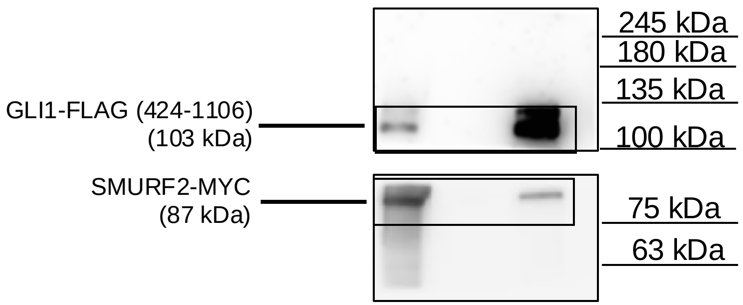

FIGURE 4C

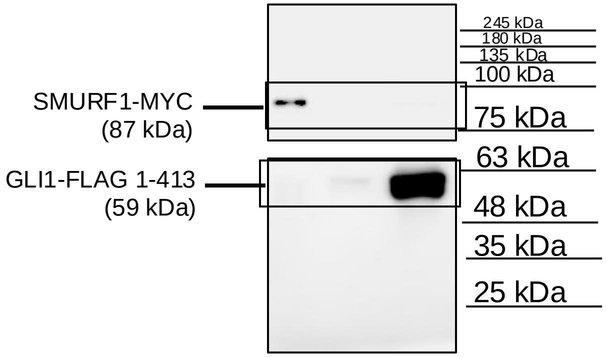

FIGURE 4D

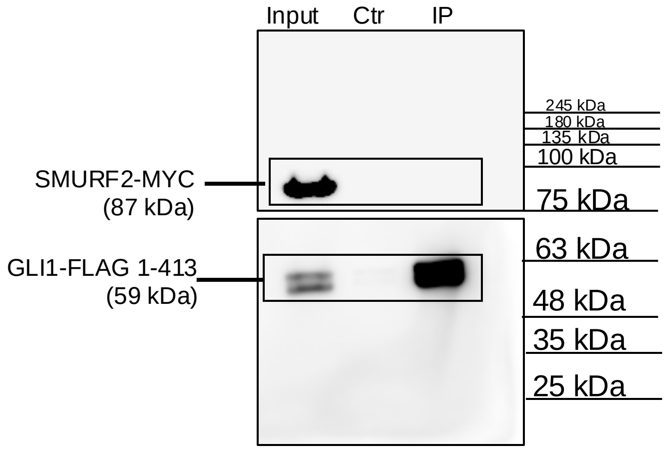

FIGURE 4E

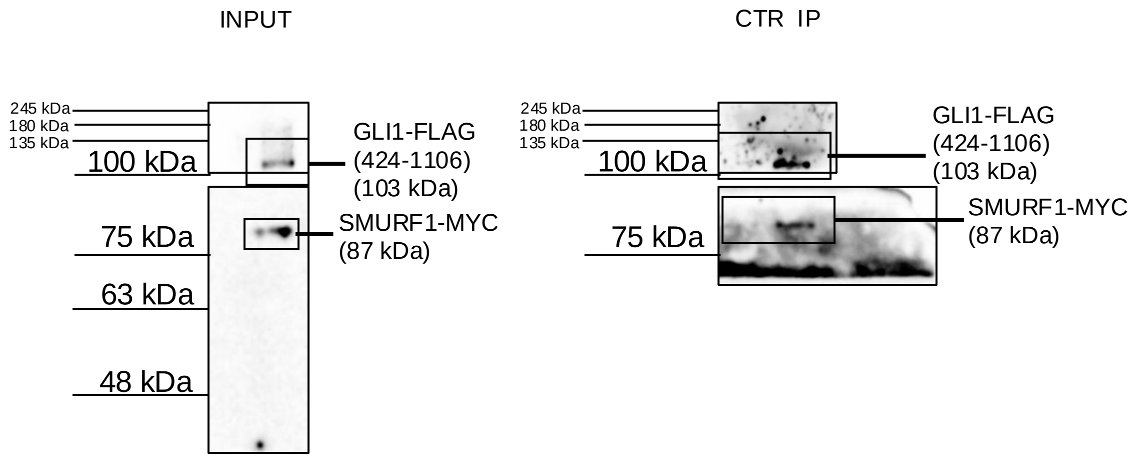

FIGURE 4F

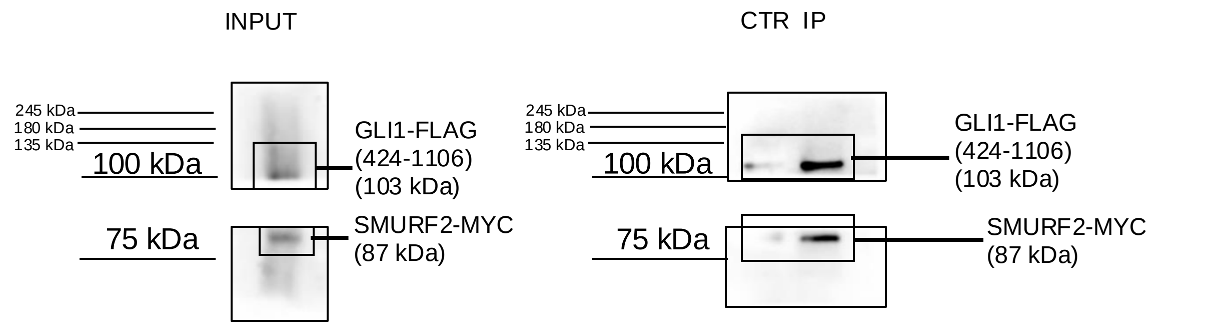

FIGURE 4G

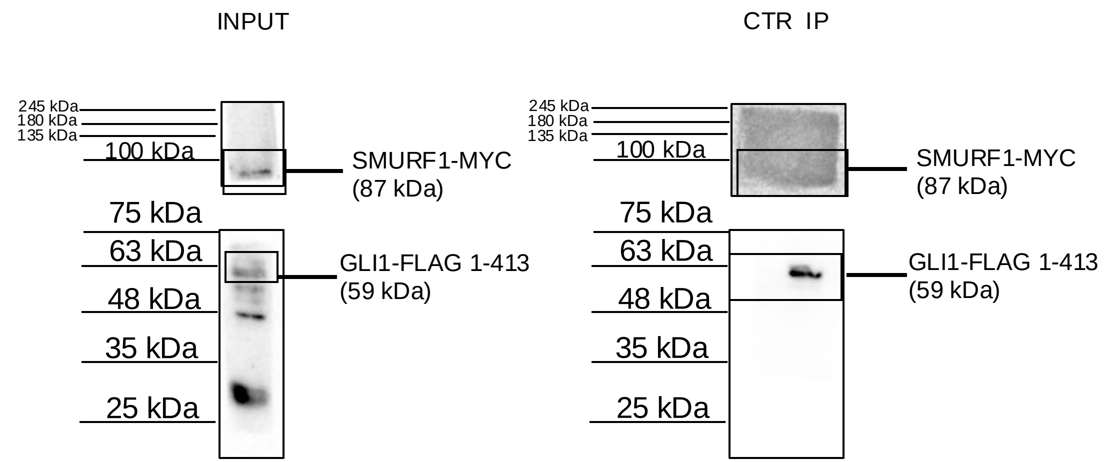

FIGURE 4H

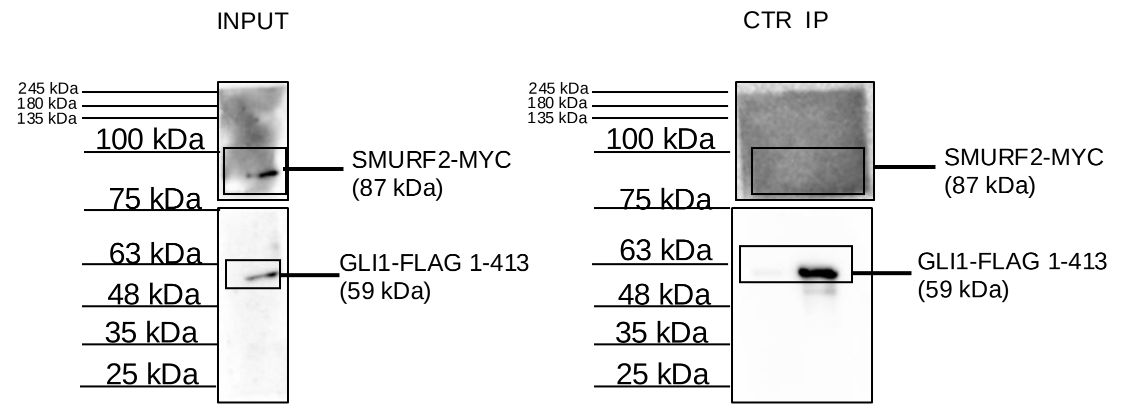

FIGURE 4I

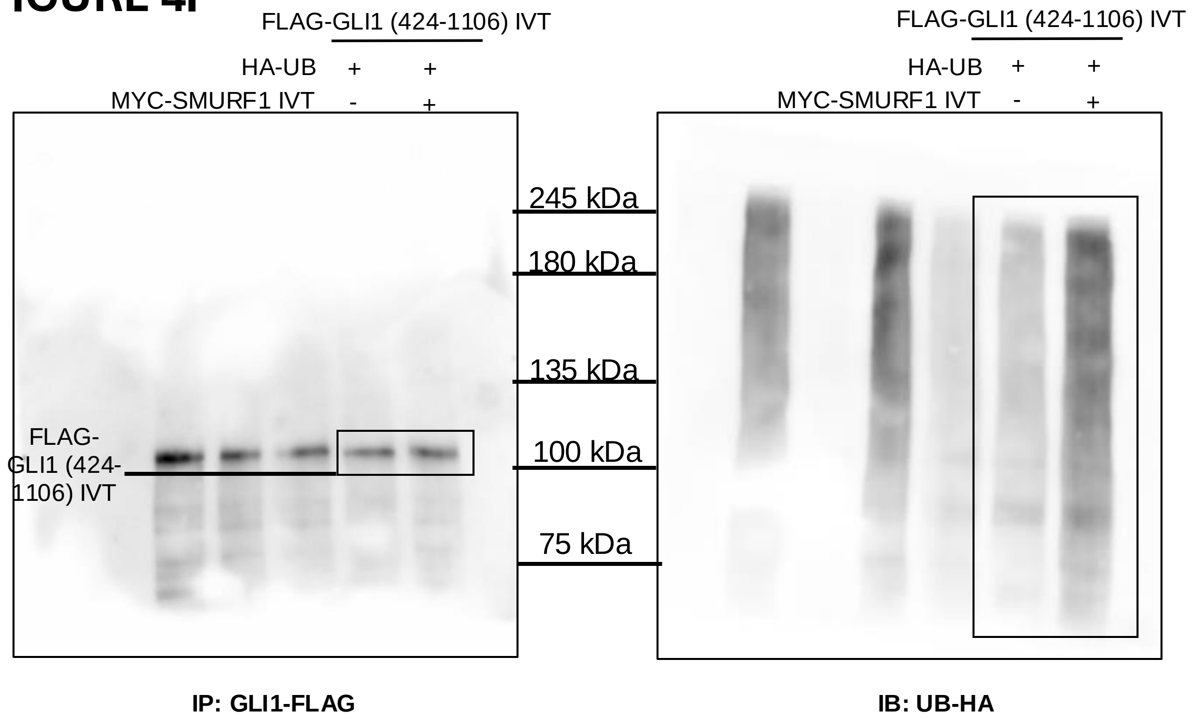

FIGURE 4J

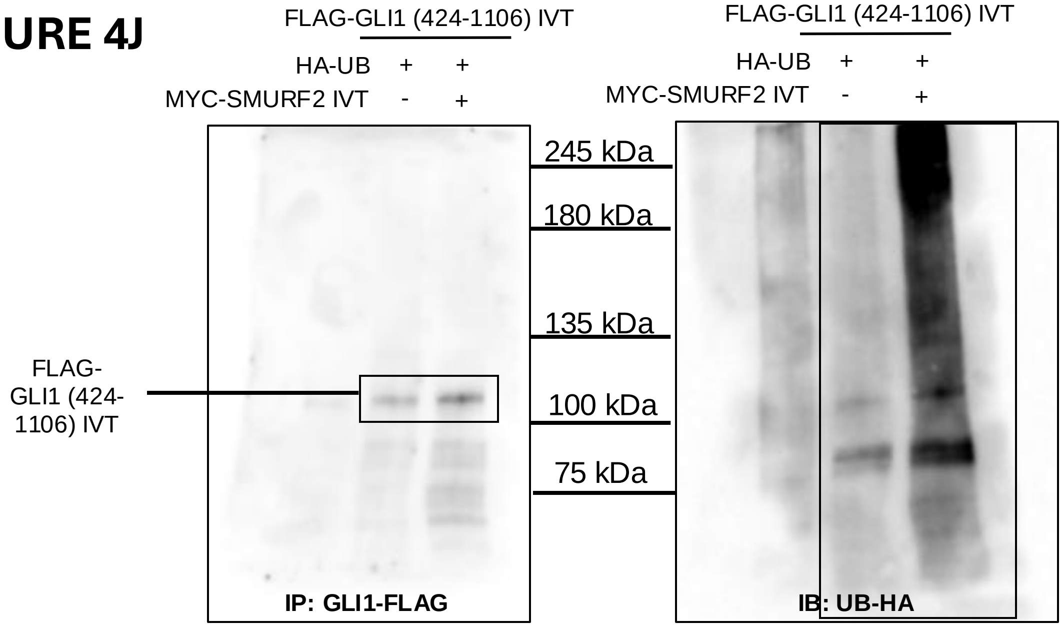

FIGURE 4K

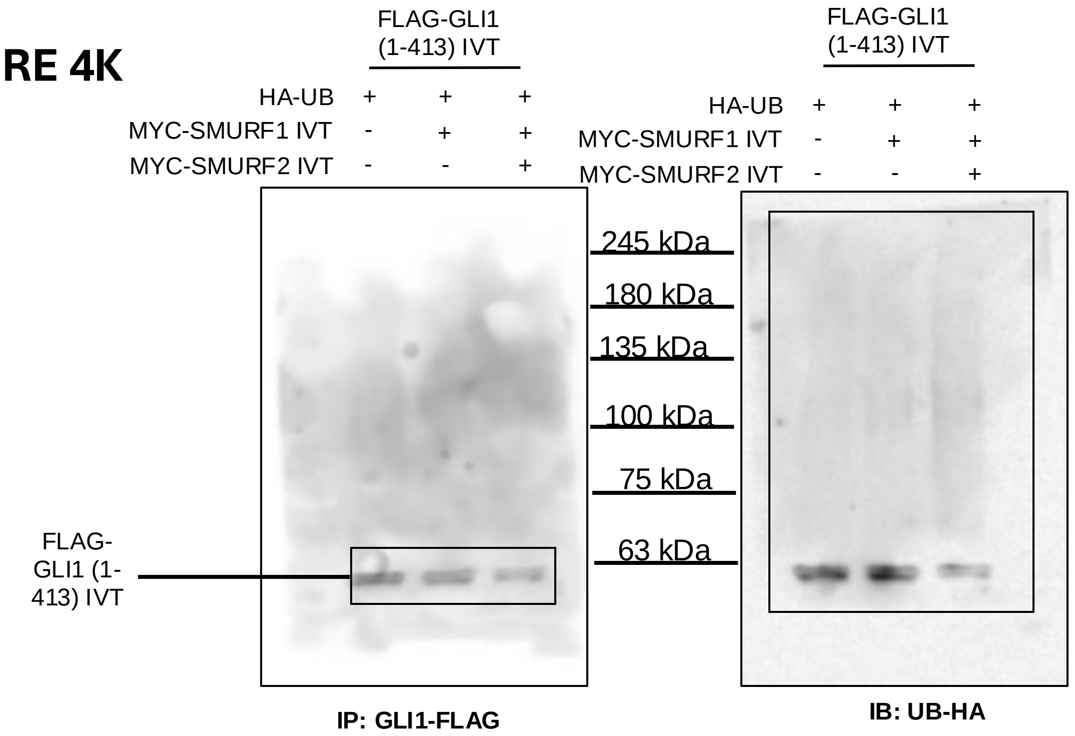

FIGURE 4L

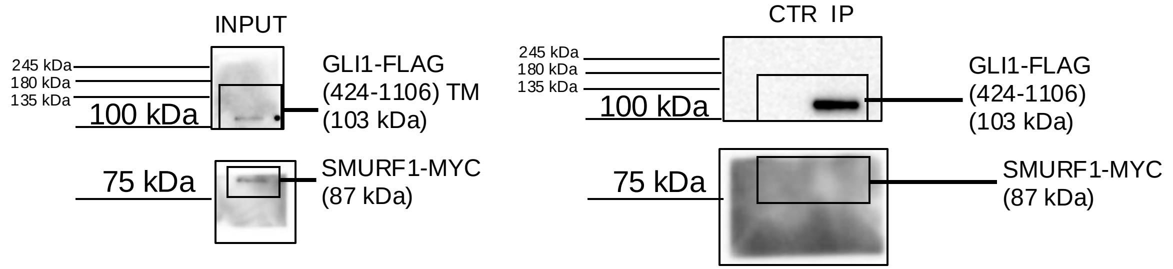

FIGURE 4M

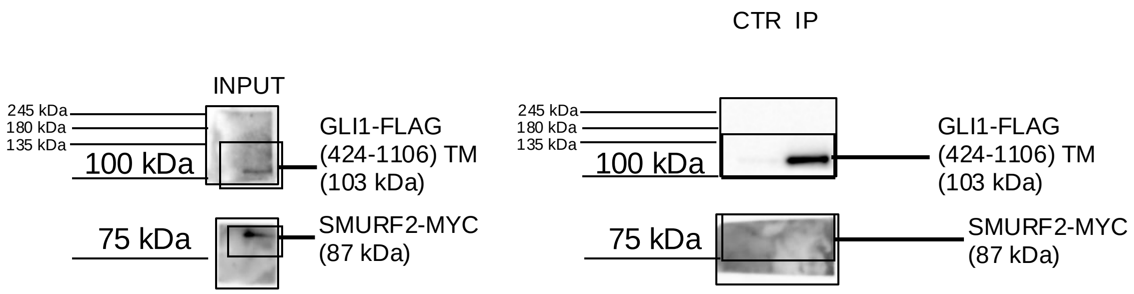

FIGURE 4N

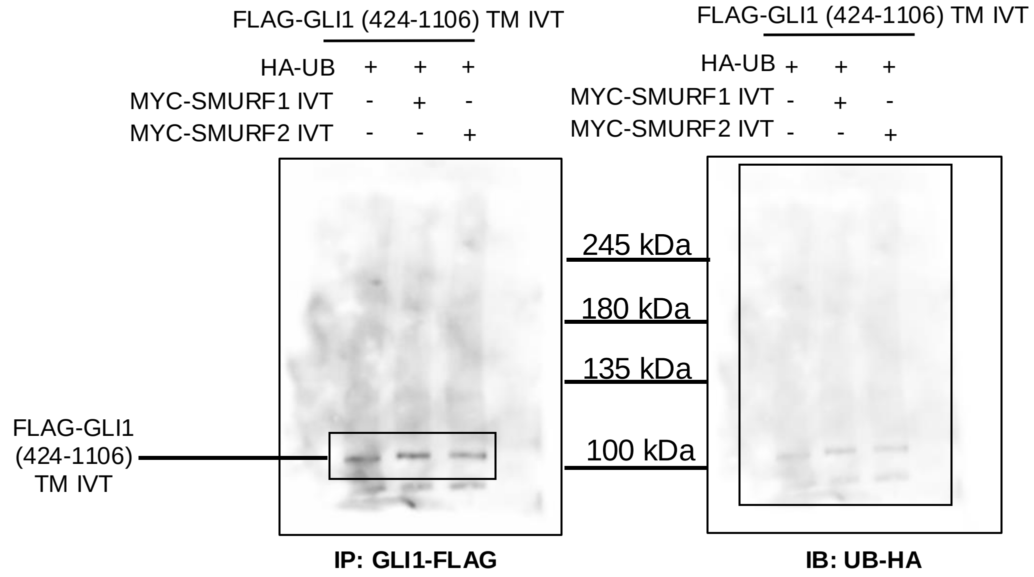

FIGURE 5A

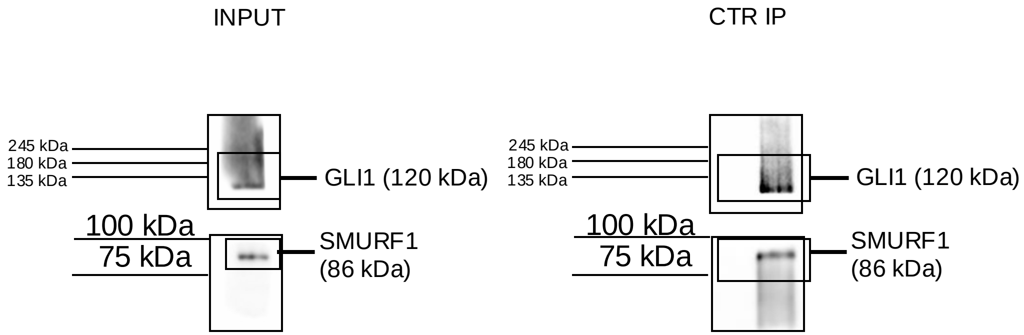

FIGURE 5B

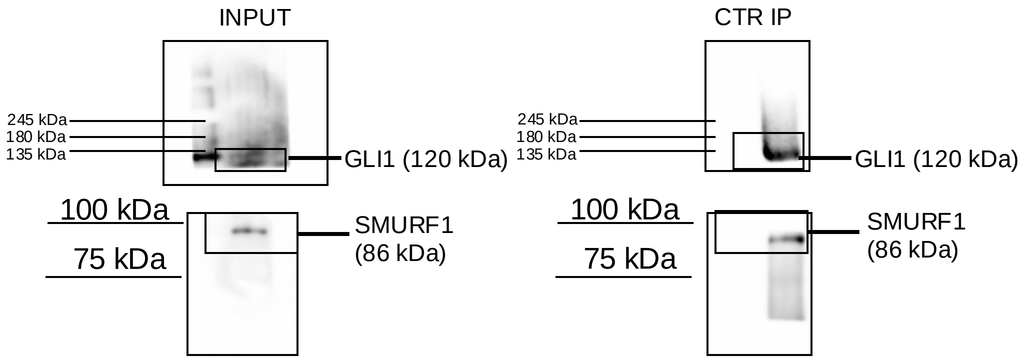

FIGURE 5C

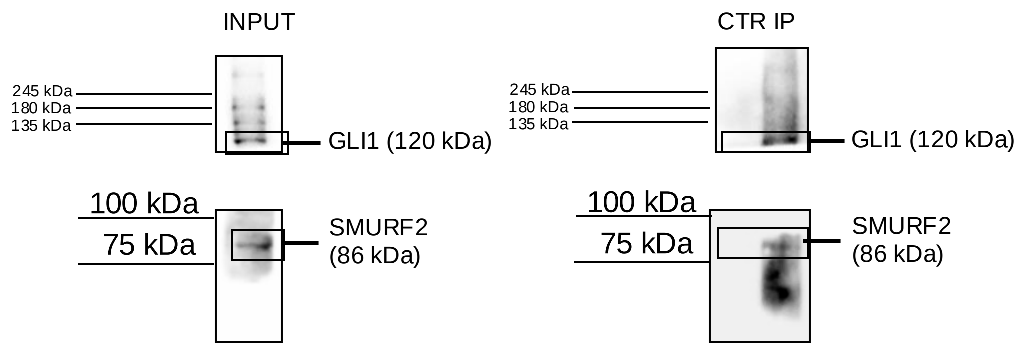

FIGURE 5D

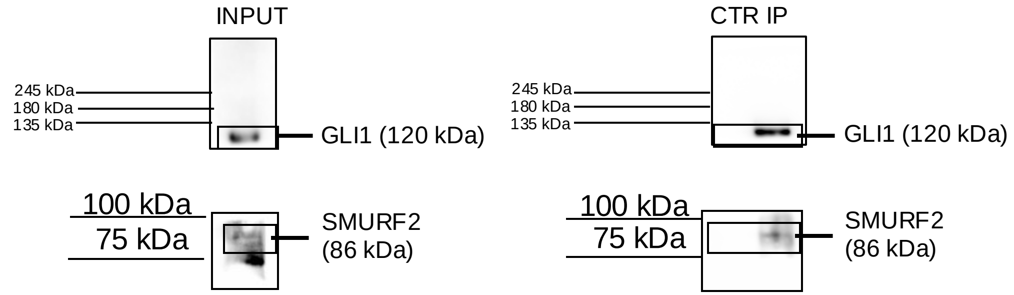

FIGURE 5E

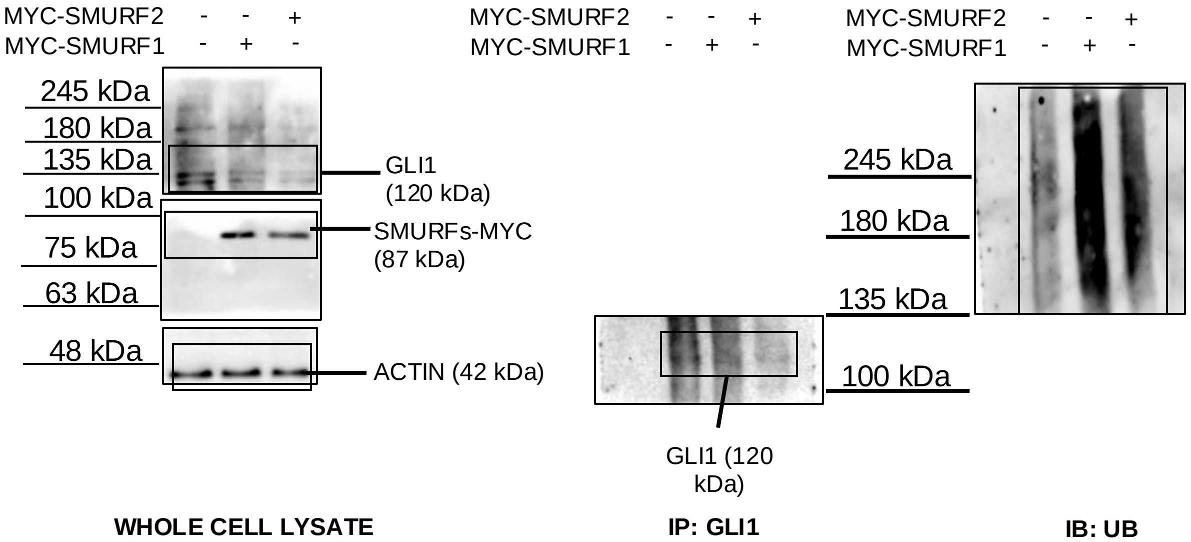

FIGURE 5F

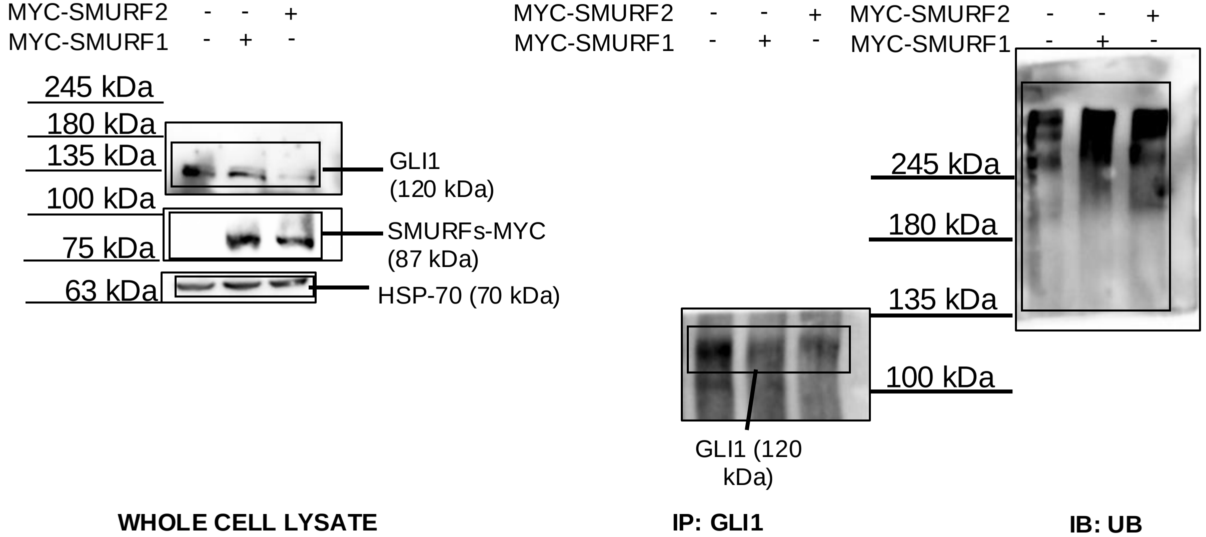

FIGURE 5G

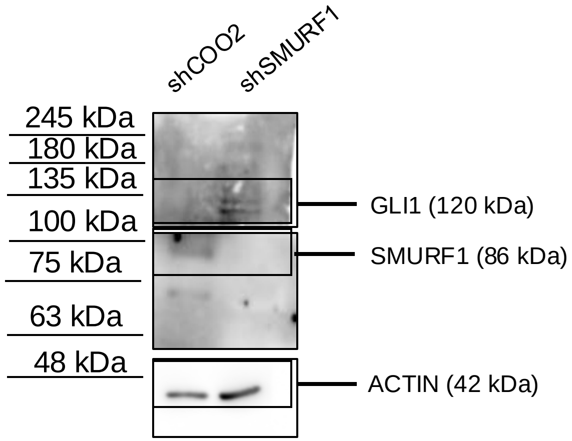

FIGURE 5H

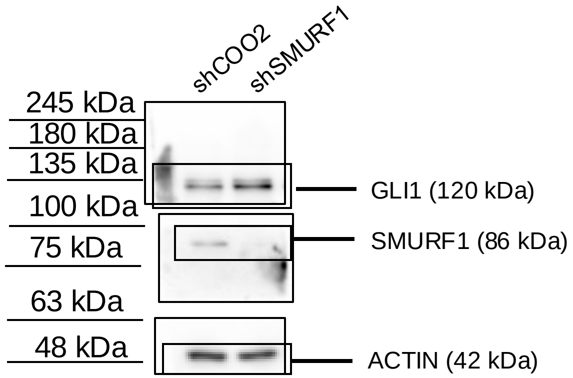

FIGURE 5I

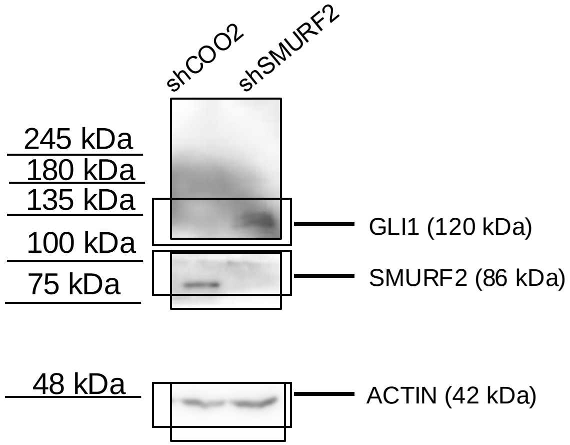

FIGURE 5J

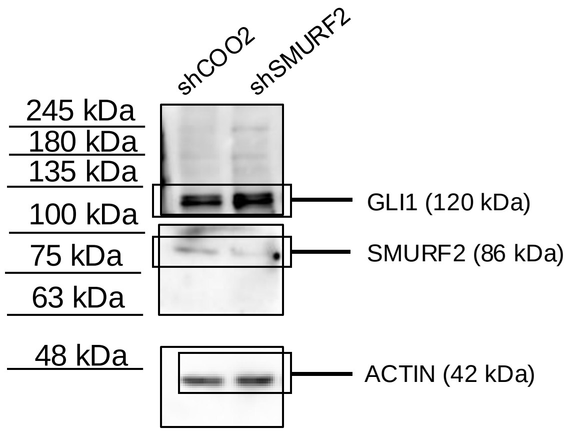

FIGURE 5K

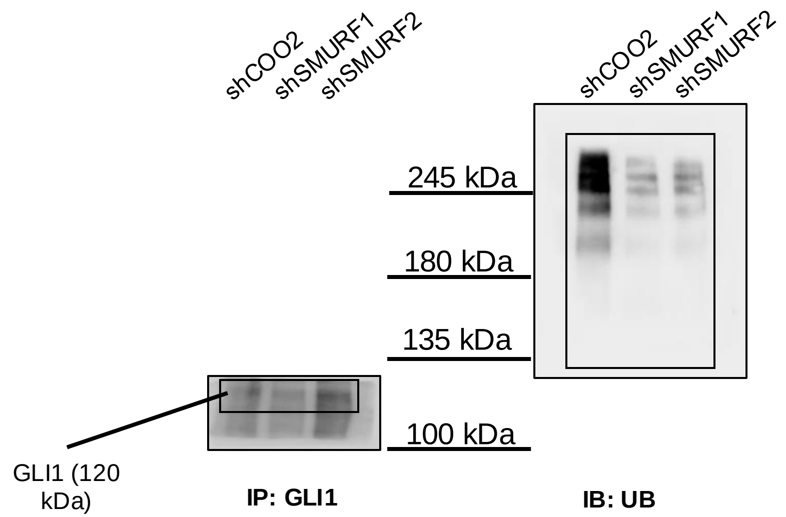

FIGURE 5L

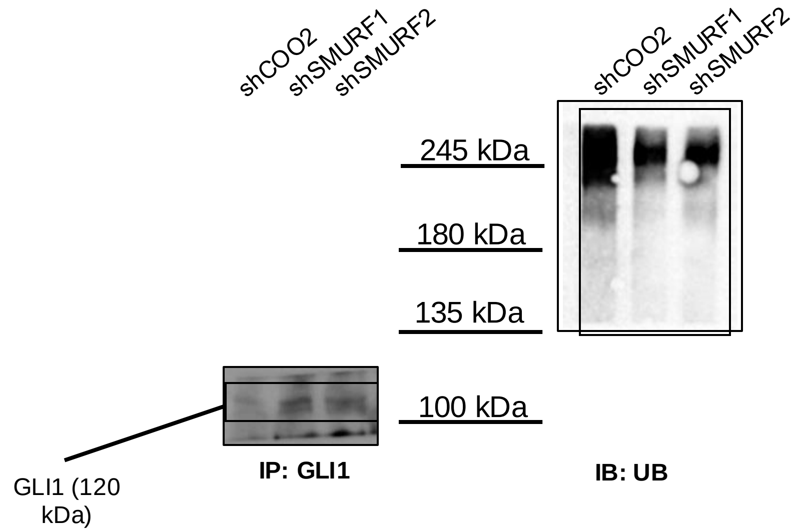

SUPPLEMENTARY S2A

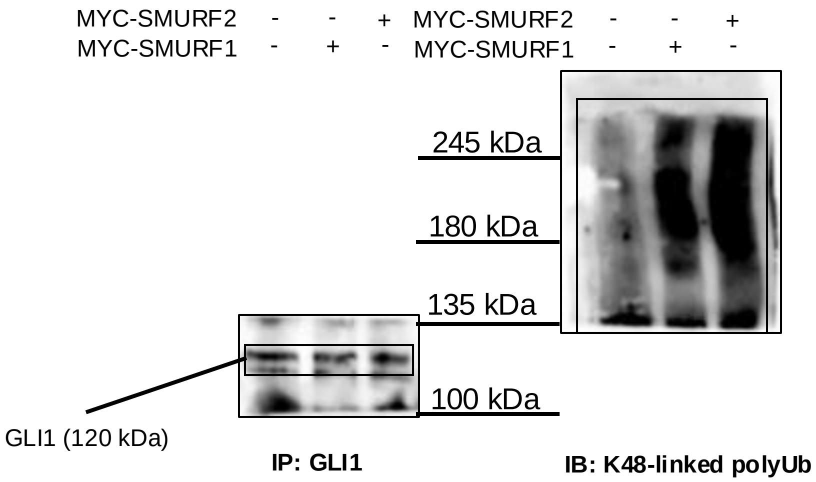

SUPPLEMENTARY S2B

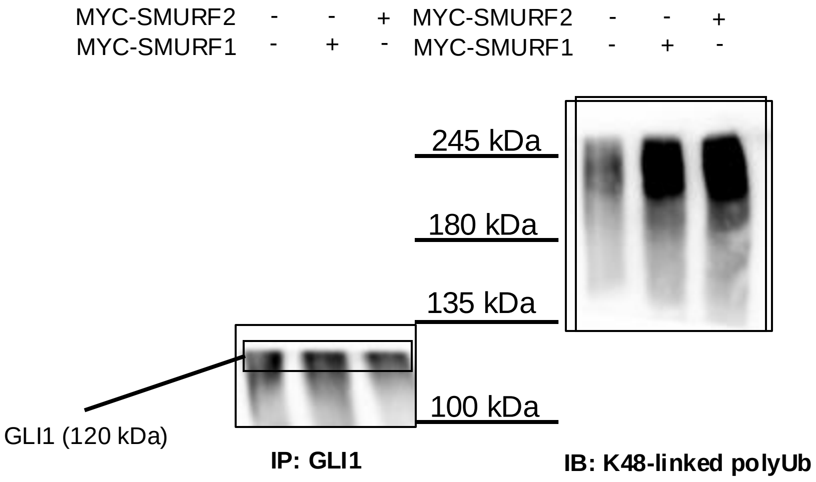

SUPPLEMENTARY 3A

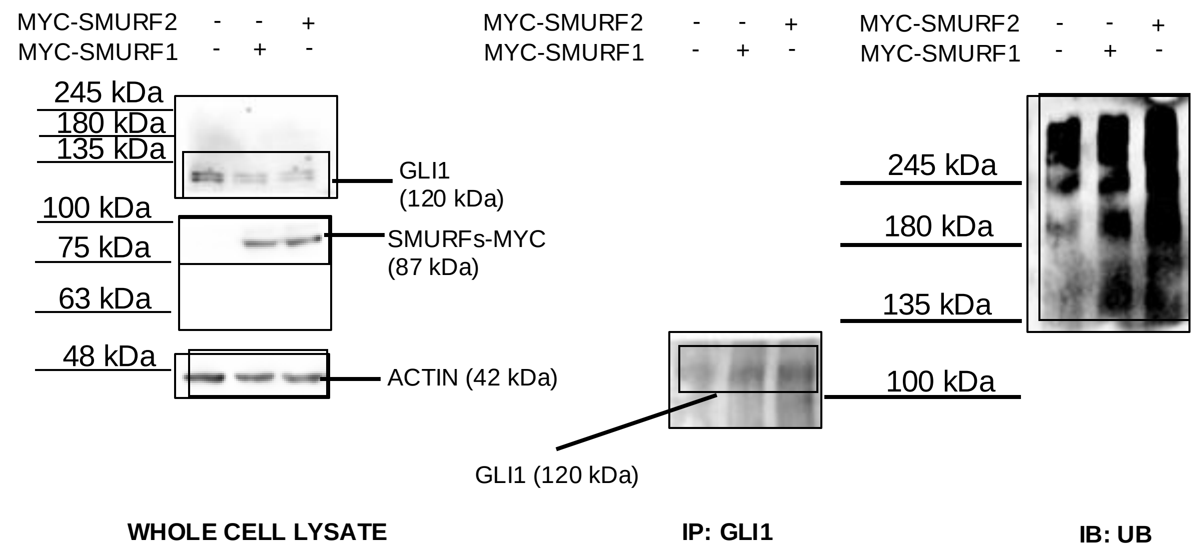

SUPPLEMENTARY 3B

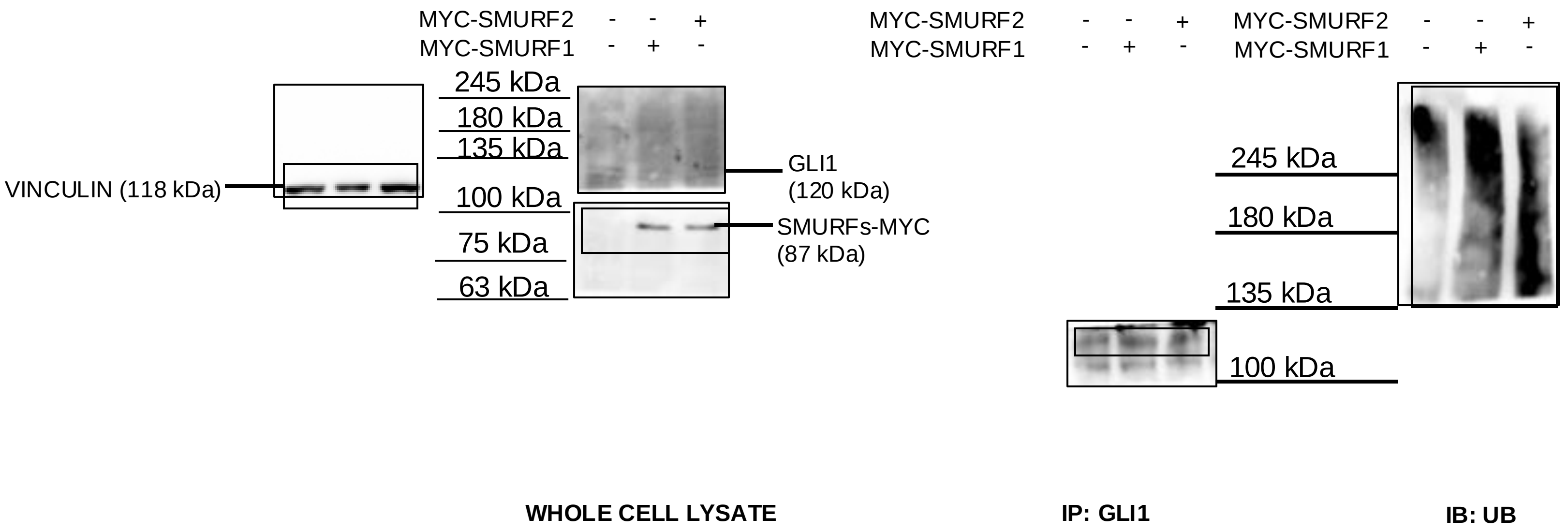

SUPPLEMENTARY 6B

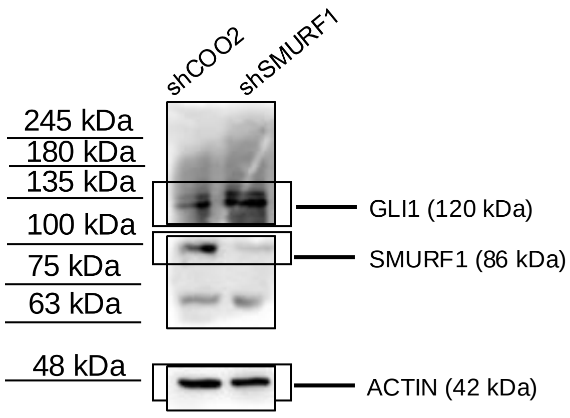

SUPPLEMENTARY 6C

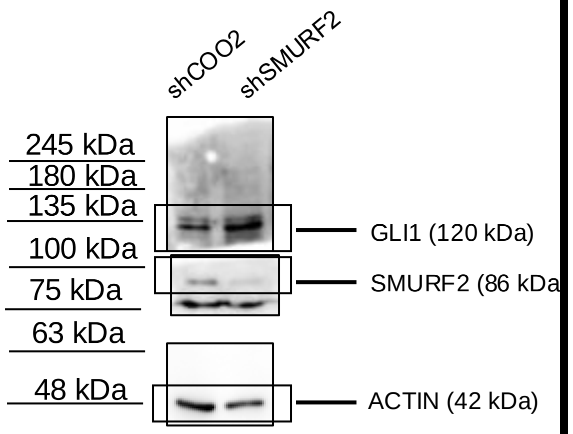

SUPPLEMENTARY S8A

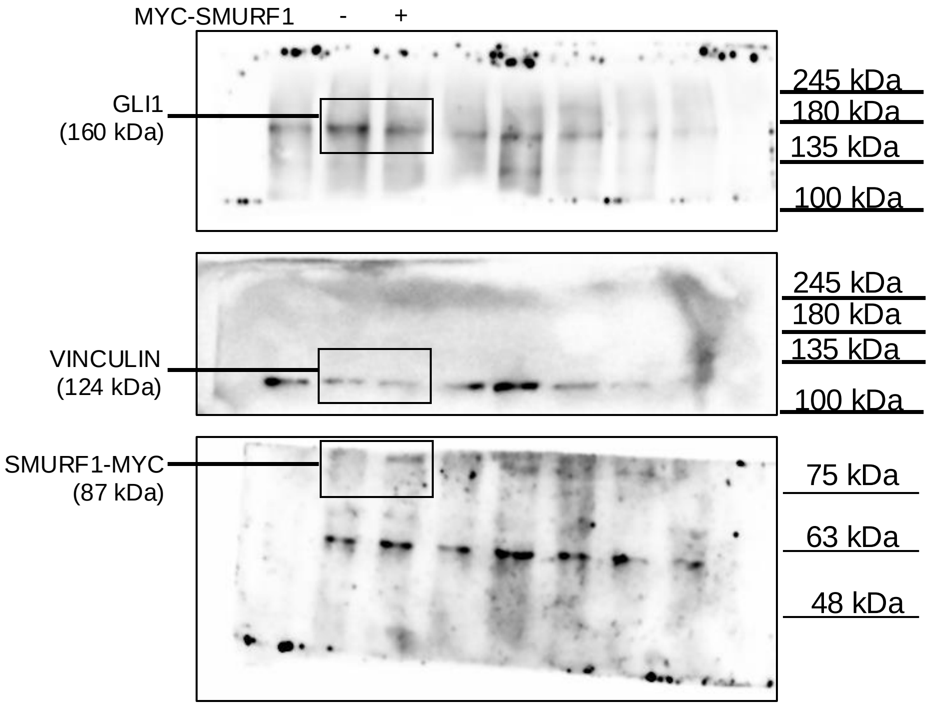

SUPPLEMENTARY S8B

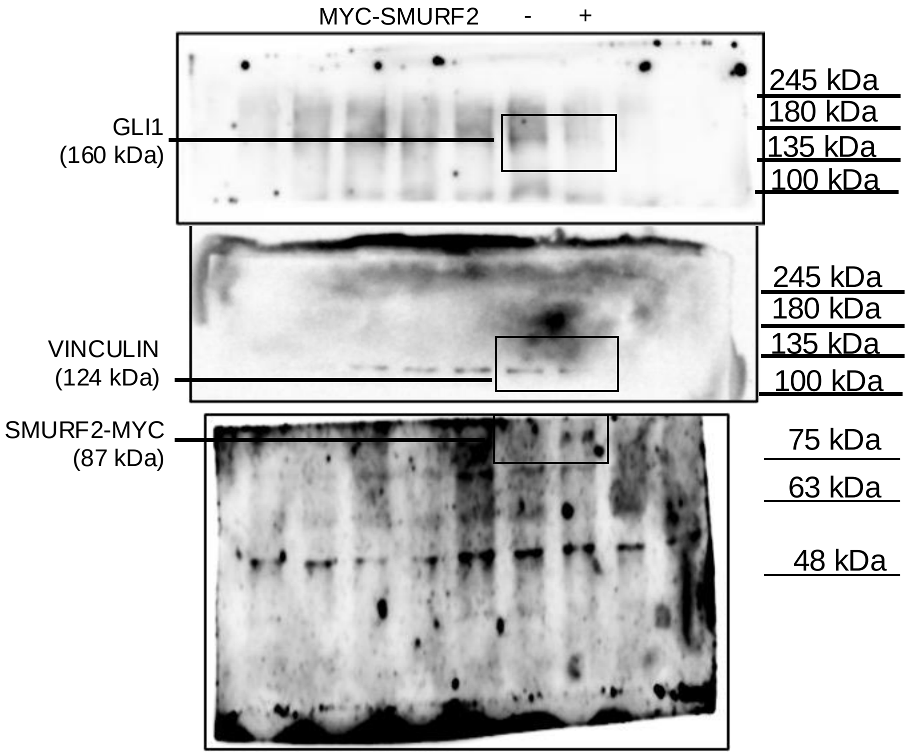

Supplement: Supplementary file 2 — Full scan of all western blot [file 41420_2024_2260_MOESM2_ESM.pdf]
